# Supplementary material for: KGR-SKATER: Spatially clustered kernel graph regression for counting processes
Source: PLoS One. 2026 May 20;21(5):e0348787. doi: 10.1371/journal.pone.0348787 (PMC13189423; doi:10.1371/journal.pone.0348787)
Supplement: S1 Appendix — (PDF) [file pone.0348787.s001.pdf]

S1 Appendix for KGR-SKATER: Spatially Clustered Kernel Graph Regression for Counting Processes

Jeffrey Wu[1,,¤*], Gareth W. Peters[1,¤,*], Alex Franks[1,¤,*],

1 Department of Statistics & Applied Probability, UCSB, Santa Barbara, California, USA

¤5607 South Hall Santa Barbara, CA 93106-2014, USA
* jeffreywu@pstat.ucsb.edu,garethpeters@pstat.ucsb.edu,afranks@pstat.ucsb.edu

S1: Application study data description

This appendix provides an expanded discussion of the datasets used in the application study, including sources, processing steps, and methodological considerations. It also includes some empirical and visual summaries of certain variables.

S1.1 Social deprivation index (SDI) scores

In [1], a group of researchers from the Society of Actuaries (SoA) examined the trends in mortality across socioeconomic groups. To do this, they constructed the Socioeconomic Index Score (SIS) as a composite measure of socioeconomic disadvantage or deprivation, hence why it is referred to as a social deprivation score (SDI) in the main paper. Building upon previous studies, it aggregates 11 carefully chosen socioeconomic subindices from the American Community Survey (ACS) into this SDI score for all counties in the US from 1982 to 2019. The 11 subindices are: 1) Percentage of the population aged 25 and over with less than 9 years of education, 2) Percentage of the population aged 25 and over with at least 4 years of college education, 3) Percentage of the population aged 16 and over employed in a white collar occupation, 4) Unemployment rate for the population 16 years and over, 5) Median household income adjusted for local housing costs, 6) Ratio of the average household income in the lowest quintile to the average household income in the highest quintile, 7) Percentage of the population below the federal poverty threshold, 8) Median home value for owner occupied units, 9) Median gross rent for rental units, 10) Percentage of housing without a telephone, and 11) Percentage of housing without complete plumbing.

As in previous studies, the final SDI score is constructed using Principal Component Analysis (PCA) on the 11 socioeconomic variables presented above. First, all variables are normalized by subtracting the mean and dividing by the standard deviation to ensure comparability across different measurement units. The standardized values are then weighted by their corresponding coefficients from the first principal component (PC1), and the weighted values are summed for each county. The resulting scores are further standardized to have a mean of 100 and a standard deviation of 20. This final standardized index represents the social deprivation for each county and year.

This SoA report comes with an Excel file containing the 11 subindices and SDI scores for every county in the US, from which the scores for California from 2010-2019 were extracted for the application study. Heatmaps of SDI from 2014-2019 can be found below.

Fig S1.1. Heatmaps of SDI scores for the counties of California from 2014-2019. County boundary shapefiles obtained from the US Census Bureau (`https://catalog.data.gov/dataset/tiger-line-shapefile-2016-state-california-current-place-state-based`). These are in the public domain. Maps were generated by the authors using R packages (*maps, sf, ggplot2*). Notice that there is a little variation over the years, but much more so across different regions.

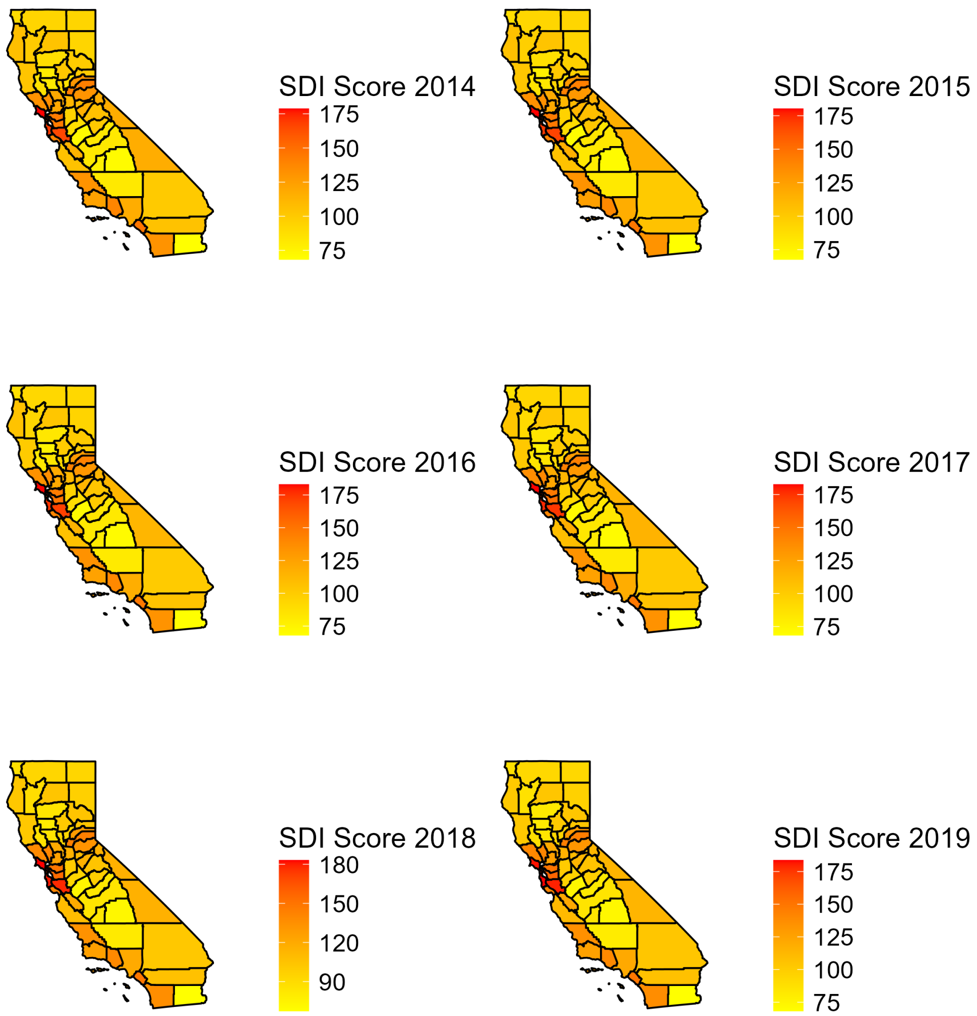

Before feeding this data into the spatial clustering step, it has to be scaled. Then, it can be used to create spatial clusters, and eventually, estimate a spatial dependence structure. While other variables, such as the response variable itself, could be used to

estimate a spatial dependence structure of respiratory related mortality, based on the findings of this report, it is reasonable to assume that variation in socioeconomic status across the counties of California can explain most of the spatial variation in mortality. This is why the SKATER and *huge* steps were performed on SDI in the application study. See S4 Appendix for discussion of how the number of clusters to be created was decided upon and S4 Appendix for discussion of how the composite SDI score, as opposed to the subindices or a set of the subindices, was chosen.

S1.2 EPA station air pollutant measurements

The US Environmental Protection Agency (EPA) provides an AQI to access their Air Quality System database which can be found at [2]. Using this API, daily pollutant concentration measurements were extracted for seven pollutants (Lead, CO, SO2, NO2, O3, PM10, PM2.5) at multiple EPA monitoring stations across California. Each county does not necessarily have an monitoring station within its borders, so a set of stations that produce a reasonable spatial coverage of California had to be carefully assembled (see S2 Appendix for details). Given a set of 5-20 monitoring stations for each county and year between 2014 and 2019, data is queried for each station using the EPA's AQS function. This function only allows for a maximum of four pollutants/parameters to be queried at once for a single year, so two calls to the function must be made for each year.

The data that is acquired from these stations are daily averages from periodic measurements made by the station throughout the day. It is not guaranteed that the data from every station is reliable, so a filter (described in S2 Appendix) was applied to remove data from stations that were missing measurements for too many days out of the year and stations that exhibited too many outlier measurements in a row. With the remaining station data (for every pollutant) for each county, a monthly median was calculated to convert the time series to the correct resolution. Then, a population weighted mean was calculated to transform the monthly time series covariates for a group of counties to the surrogate variables defined in the paper, a cluster level, monthly time series for each of the seven pollutants.

Each pollutant measurement comes with an air quality index (AQI) standardized measurement for that specific pollutant. Each pollutant has a different standardizing equation, but if they are all standardized, they can just be compared with each other to identify the maximum. It is stated by the EPA that the reported AQI level reported for each day is the maximum of the AQI values reported for each of the pollutants, so the maximum AQI among the different standardized measurements for a given county and a given month was used to represent the AQI.

Finally, each cluster level time series was inspected to ensure stationarity. The Lead time series needed to be differenced at one lag, the other pollutant time series were already stationary. With all of the air quality data now processed to the monthly cluster level it can be used as surrogate covariates in the construction of a time series regression kernel matrix for the KGR-SKATER models. The assumption being that temporal variation in these air pollutant covariates can explain most of the temporal variation in mortality, a notion supported by [3]. Plotting the time series for every pollutant for each cluster would have introduced too many plots so instead, heatmaps for AQI for each county from July to December 2014 have been included below to give a sense of the spatiotemporal variation in air quality:

Fig S1.2. Heatmaps of AQI for the counties of California from 2014-2019. County boundary shapefiles obtained from the US Census Bureau (`https://catalog.data.gov/dataset/tiger-line-shapefile-2016-state-california-current-place-state-based`). These are in the public domain. Maps were generated by the authors using R packages (*maps, sf, ggplot2*). Notice that there is more variation over time than between different locations. The blue dots represent the station locations used to create the air quality covariates for each county.

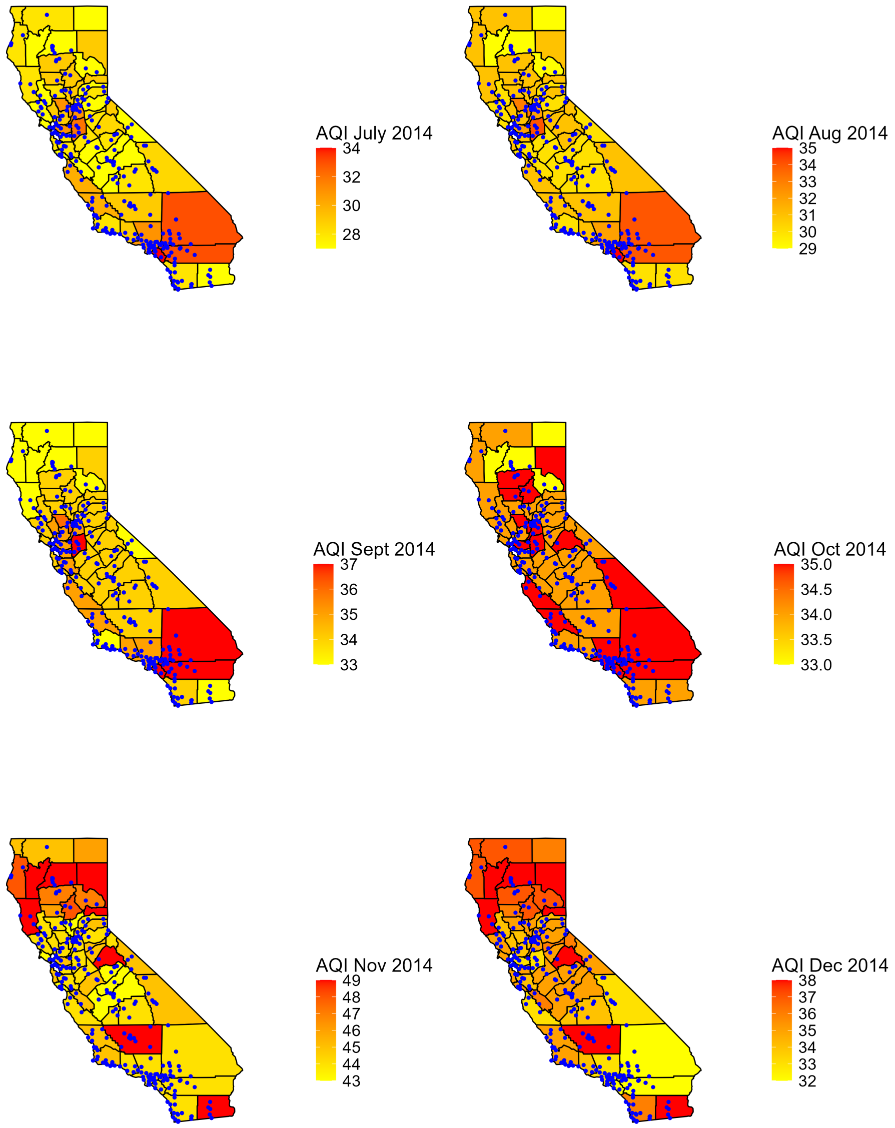

S1.3 Respiratory related mortality counts

The California Vital Data (Cal-ViDa) query tool allows one to access California's birth and death data courtesy of [4]. CalViDa provides counts of death at the state, county, or city level and is able to stratify these counts by age group and cause of death. For the main paper's application study, the goal was to model the total number of respiratory related deaths and so only deaths classified under chronic lower respiratory diseases and influenza or pneumonia cause of death were retained. These were the only two categories that were related to the respiratory system. The death counts attributable to these two causes were to be added together however, before these counts could be added together, the censoring of small counts (between one and ten) had to be addressed. See S3 Appendix for an explanation of how these censored counts were imputed with the Expectation Maximization (EM) Algorithm.

After adding the two causes of death categories together, the resulting dataset contains death counts by age group, which might be useful for future investigations. Including age group as a covariate in the model could add some potentially interesting nuance to the findings, but this is not pertinent to the main objective of demonstrating the utility of the KGR-SKATER modeling framework. So death counts were also added up across age groups to get a total number of respiratory related deaths (attributable to the two causes of death mentioned above) for each county for each month between 2014 and 2019. The heatmaps below show the population and respiratory related death rate per county for each year to once again give a sense of the spatiotemporal variation in the observation process:

Fig S1.3. Heatmaps of respiratory-related mortality rate for the counties of California from July to December 2014. County boundary shapefiles obtained from the US Census Bureau (`https://catalog.data.gov/dataset/tiger-line-shapefile-2016-state-california-current-place-state-based`). These are in the public domain. Maps were generated by the authors using R packages (*maps, sf, ggplot2*). There appears to be a little variation across the different counties over the months.

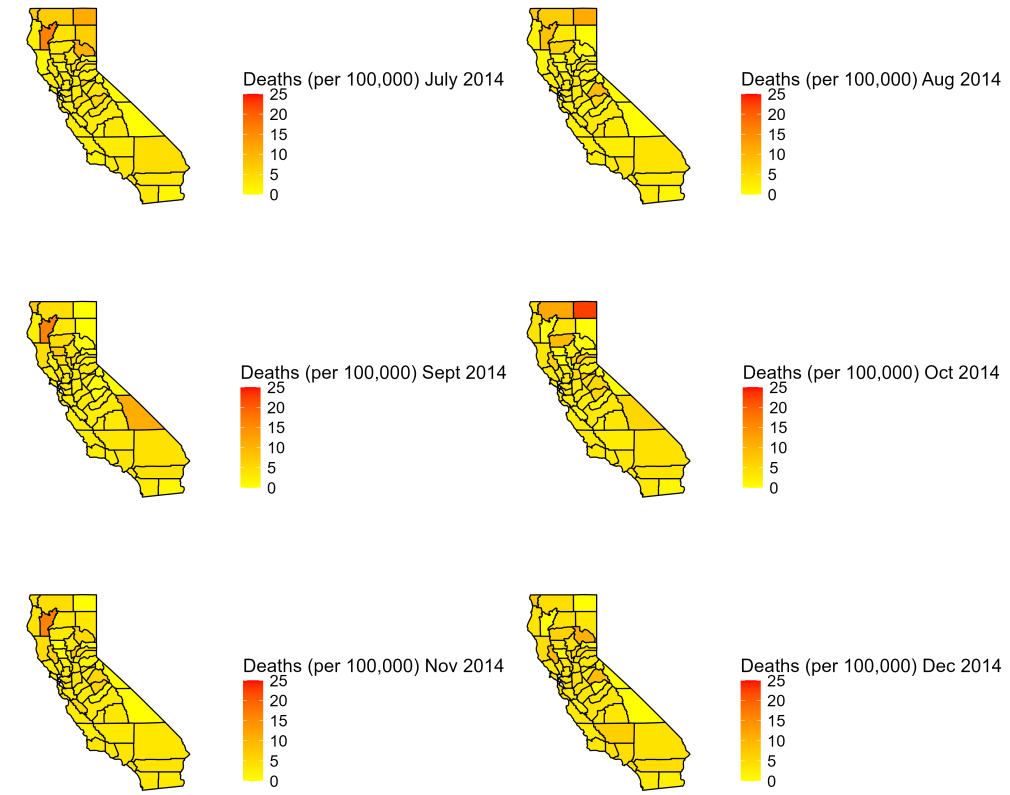

References

1. Barbieri M. Mortality by Socioeconomic Category in the United States; 2020. Available from: https://www.soa.org/resources/research-reports/2020/us-mort-rate-socioeconomic/#report.

2. Agency UEP. Air Quality System Data Mart; 2024. Accessed February 11, 2024. Available from: https://www.epa.gov/outdoor-air-quality-data.

3. Chen YH, Mukherjee B, Berrocal VJ. Distributed Lag Interaction Models with Two Pollutants. 2019;68(1):79-97. Available from: https://www.ncbi.nlm.nih.gov/pmc/articles/PMC6328049/. doi:10.1111/rssc.12297.

4. State of California DoPH. California Vital Data (Cal-ViDa), Death Query; 2024. Accessed: 2023-06-18. Available from: https://cal-vida.cdph.ca.gov/.
