# Supplementary material for: KGR-SKATER: Spatially clustered kernel graph regression for counting processes
Source: PLoS One. 2026 May 20;21(5):e0348787. doi: 10.1371/journal.pone.0348787 (PMC13189423; doi:10.1371/journal.pone.0348787)

S2 Appendix for KGR-SKATER: Spatially Clustered Kernel Graph Regression for Counting Processes

Jeffrey Wu[1,,¤*], Gareth W. Peters[1,¤,*], Alex Franks[1,¤,*],

1 Department of Statistics & Applied Probability, UCSB, Santa Barbara, California, USA

¤5607 South Hall Santa Barbara, CA 93106-2014, USA
* jeffreywu@pstat.ucsb.edu,garethpeters@pstat.ucsb.edu,afranks@pstat.ucsb.edu

S2: Procedure for obtaining air quality and pollutant measurements for each county

In addition to the restrictive nature of the EPA's air quality system (AQS) API, it is well known that the data provided by EPA measurement stations can be unreliable, so it was important to systematically query and clean the data that was obtained. The main steps were to first identify all possible stations in California measuring a given pollutant, say PM 2.5, for a given year. Then from that set of stations, identify a subset of stations that would give us the "best" spatial coverage of California; the problem being that not all counties had a station measuring each pollutant. Fig S2.1 illustrates this. A subset of stations was identified by taking the two largest cities, based on population, in each county and finding five to fifteen stations that were within a certain radius (usually 100km) of each city. This was done to ensure that, each year, each county would have a reasonable number of stations measuring air quality in that general vicinity, which would hopefully in turn lead to a reasonable spatial coverage of the entire state. Next, after pulling the data corresponding to the subset of "best" stations, a quality check function was created to filter out any stations that produced datasets that were not up to standard. It was decided that a reasonable standard for inclusion into the air quality dataset would be to have at least 240 out of the 365 possible data points in a year and not have more than 14 consecutive measurements that qualify as outliers according to the Hampel filter, which is based on median values instead of averages. Hopefully, this filters out all the problematic datasets that would otherwise be included in the calculation of the other component of the KGR-SKATER covariance structure, the kernel gram matrix $\mathbf{K}$.

Fig S2.1. Selected set of EPA station locations in California from 2014-2019 plotted over the clusters created by SKATER. County boundary shapefiles obtained from the US Census Bureau (`https://catalog.data.gov/dataset/tiger-line-shapefile-2016-state-california-current-place-state-based`). These are in the public domain. Maps were generated by the authors using R packages (*maps, sf, ggplot2*). These are the stations from which air pollutant measurement data are drawn from and used to calculate some of the gram matrices. A set of stations was obtained that provides a reasonable spatial coverage of the state and each cluster; however, note that most stations do not measure all pollutants. This means that some clusters may have stations measuring some pollutants but not others, so there are cases where data for a specific pollutant is the same across multiple clusters.

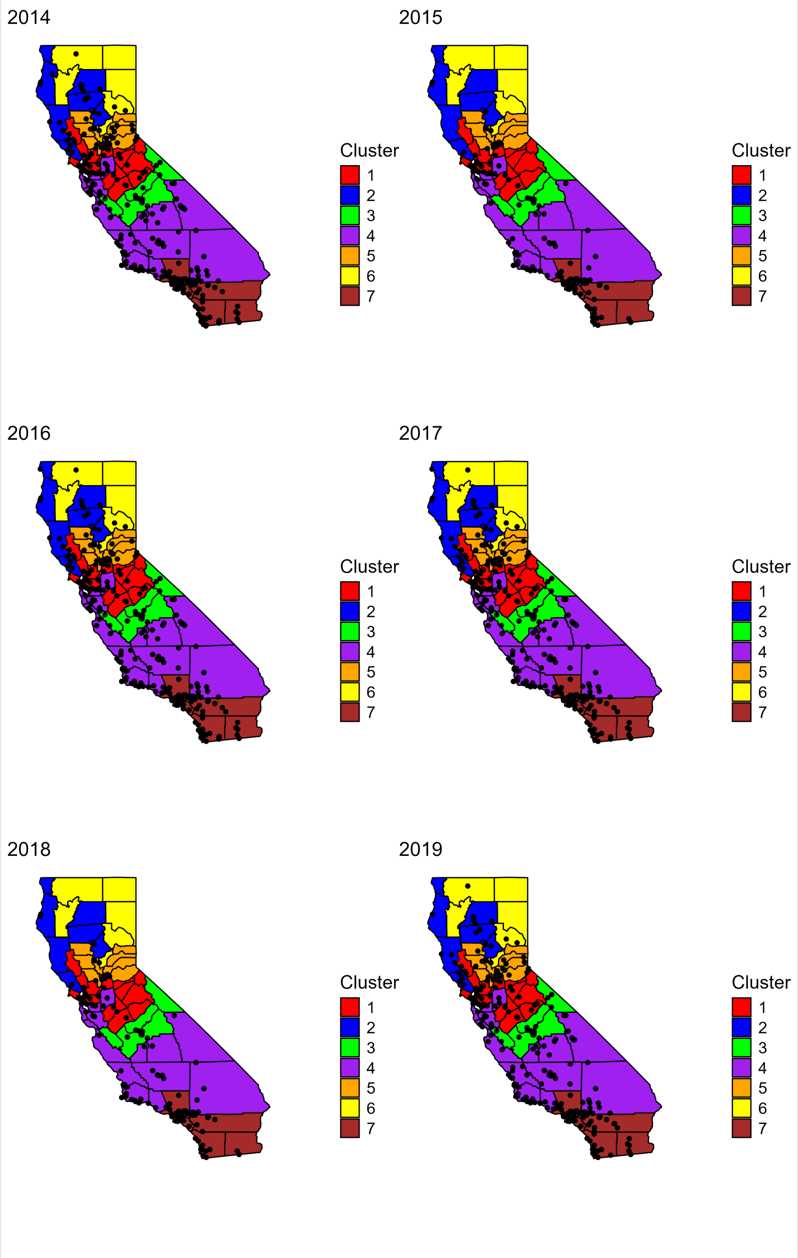

Supplement: S2 Appendix — (PDF) [file pone.0348787.s002.pdf]
