# Supplementary material for: KGR-SKATER: Spatially clustered kernel graph regression for counting processes
Source: PLoS One. 2026 May 20;21(5):e0348787. doi: 10.1371/journal.pone.0348787 (PMC13189423; doi:10.1371/journal.pone.0348787)
Supplement: S3 Appendix — (PDF) [file pone.0348787.s003.pdf]

S3 Appendix for KGR-SKATER: Spatially Clustered Kernel Graph Regression for Counting Processes

Jeffrey Wu[1,¤*], Gareth W. Peters[1,¤,*], Alex Franks[1,¤,*],

1 Department of Statistics & Applied Probability, UCSB, Santa Barbara, California, USA

¤5607 South Hall Santa Barbara, CA 93106-2014, USA
* jeffreywu@pstat.ucsb.edu,garethpeters@pstat.ucsb.edu,afranks@pstat.ucsb.edu

S3: Imputing "< 11" values in data with EM algorithm

This appendix describes the process for imputing censored small counts in the CalViDa mortality dataset using an approximate EM algorithm.

In this section, for simplicity and a bit of abuse of notation, Y_i will denote ith spatiotemporal observation where i indexes individuals by time i.e. $i = 1, 2, \ldots, n = NT$. For the EM algorithm, the rate will be modeled using a generalized linear model where the variables month (the time index), cause of death, and age group are used as predictors. So assume that $\lambda_i(\beta) = \exp(\alpha_i + \mathbf{X}_i^T \boldsymbol{\beta})$, where α_i is the offset (log of county population of the age group) and $\mathbf{X}_i$ are predictors (month, cause of death, age group).

Due to interval censoring, the exact mortality counts for some units are not observed. Let C_i be the censoring indicator such that $C_i = I(1 \le Y_i \le 10)$, i.e., the count is censored if it is between 1 and 10.

Note that C_i has marginal distribution such that $P(C_i = 1) = \sum_{j=1}^{10} \frac{\lambda_i(\boldsymbol{\beta})^j e^{-\lambda_i(\boldsymbol{\beta})}}{j!}$. The joint distribution of (C_i, Y_i) has the following probability mass function

$$P(C_i = c_i, Y_i = y_i|\boldsymbol{\beta}) = \begin{cases} \frac{\lambda_i(\boldsymbol{\beta})^{y_i} e^{-\lambda_i(\boldsymbol{\beta})}}{y_i!}, & \text{if } c_i = 1, 1 \le y_i \le 10; \\ \frac{\lambda_i(\boldsymbol{\beta})^{y_i} e^{-\lambda_i(\boldsymbol{\beta})}}{y_i!}, & \text{if } c_i = 0, y_i = 0 \text{ or } y_i > 10; \\ 0, & \text{otherwise.} \end{cases}$$

Let $\mathbf{W}$ denote the set of indices for observations that are censored, i.e. $\mathbf{W} = \{i : C_i = 1\}$. Note that for unit $i \in \mathbf{W}$, $(C_i = 1)$; on the other hand, for unit $i \notin W$ $(C_i = 0, Y_i = y_i)$ is observed with $y_i = 0$ or $y_i > 10$. Hence, assuming independence across units, the likelihood of the observed data $\mathbf{C} = (C_1, C_2, \ldots C_n)$ and $\mathbf{Y}_{obs} = (Y_i = y_i, i \notin W)$ is

$$\begin{aligned} L[\boldsymbol{\beta}; &(C_1, C_2, \ldots C_n), (Y_i = y_i, i \notin \mathbf{W})] \\ &= \prod_{i \in \mathbf{W}} P(C_i = 1) \prod_{i \notin \mathbf{W}} [P(C_i = 0, Y_i = y_i) I(y_i = 0 \text{ or } y_i > 10)] \\ &= \prod_{i \in \mathbf{W}} \left[\sum_{j=1}^{10} \frac{\lambda_i(\boldsymbol{\beta})^j e^{-\lambda_i(\boldsymbol{\beta})}}{j!} \right] \prod_{i \notin \mathbf{W}} \left[\frac{\lambda_i(\boldsymbol{\beta})^{y_i} e^{-\lambda_i(\boldsymbol{\beta})}}{y_i!} I(y_i = 0 \text{ or } y_i > 10) \right]. \end{aligned}$$

Given the nature of this complicated likelihood, the EM algorithm is used to get the MLEs of the unknown values $\boldsymbol{\beta}$.

First, for the complete data that includes $\mathbf{C} = (C_1, C_2, \dots C_n)$, $\mathbf{Y}_{obs} = (Y_i = y_i, i \notin \mathbf{W})$ and censored outcomes $\mathbf{Y}_{missing} = (Y_i = y_i^*, i \in \mathbf{W})$, the full likelihood is given by:

$$\begin{aligned}
L[\boldsymbol{\beta}; &(C_1, C_2, \dots C_n), (Y_i = y_i, i \notin \mathbf{W}), (Y_i = y_i^*, i \in \mathbf{W})] \\
&= P\left[(C_1, C_2, \dots C_n), (Y_i = y_i, i \notin \mathbf{W}), (Y_i = y_i^*, i \in \mathbf{W}) | \boldsymbol{\beta}\right] \\
&= \prod_{i \in \mathbf{W}} \left[P(C_i = 1, Y_i = y_i^*) I(1 \le y_i^* \le 10)\right] \\
&\quad \prod_{i \notin \mathbf{W}} \left[P(C_i = 0, Y_i = y_i) I(y_i = 0 \text{ or } y_i > 10)\right] \\
&= \prod_{i \in \mathbf{W}} \left[\frac{\lambda_i(\boldsymbol{\beta})^{y_i^*} e^{-\lambda_i(\boldsymbol{\beta})}}{y_i^*!} I(1 \le y_i^* \le 10)\right] \\
&\quad \prod_{i \notin \mathbf{W}} \left[\frac{\lambda_i(\boldsymbol{\beta})^{y_i} e^{-\lambda_i(\boldsymbol{\beta})}}{y_i!} I(y_i = 0 \text{ or } y_i > 10)\right].
\end{aligned}$$

Since all indicator functions in the likelihood equal 1 on the support, the corresponding log likelihood is given by:

$$\begin{aligned}
\log L(&\boldsymbol{\beta}\,; \mathbf{C}, \mathbf{Y}_{obs}, \mathbf{Y}_{missing}) \\
&= \sum_{i \in W} \left[y_i^* \log(\lambda_i(\boldsymbol{\beta})) - \lambda_i(\boldsymbol{\beta}) - \log(y_i^*!)\right] + \\
&\quad \sum_{i \notin W} \left[y_i \log(\lambda_i(\boldsymbol{\beta})) - \lambda_i(\boldsymbol{\beta}) - \log(y_i!)\right]
\end{aligned}$$

Now, this log likelihood function is carried into the Expectation or E step and then Maximization or M step of the EM algorithm. These two steps, which are described below, are iteratively executed until the estimates of $\boldsymbol{\beta}$ have converged.

In the E step, the objective is to calculate $Q(\boldsymbol{\beta}|\boldsymbol{\beta}^{(t)})$. This is achieved by taking the expected value of the log likelihood function with respect to the current conditional distribution of $\mathbf{Y}_{missing}$ given $\mathbf{C}, \mathbf{Y}_{obs}$ and the current estimates of the parameters $\boldsymbol{\beta}^{(t)}$ i.e. calculating $E_{\mathbf{Y}_{missing}|\mathbf{C}, \mathbf{Y}_{obs}, \boldsymbol{\beta}^{(t)}} \log L(\boldsymbol{\beta}\,; \mathbf{C}, \mathbf{Y}_{obs}, \mathbf{Y}_{missing})$. Here, t represents the current iteration of the algorithm.

Note that for $i \in \mathbf{W}$ and $1 \le j \le 10$,

$$P\left[Y_i = j | \mathbf{C}, \mathbf{Y}_{obs}, \boldsymbol{\beta}^{(t)}\right] = P[Y_i = j | C_i = 1, \boldsymbol{\beta}^{(t)}] = \frac{\frac{\lambda_i(\boldsymbol{\beta}^{(t)})^j e^{-\lambda_i(\boldsymbol{\beta}^{(t)})}}{j!}}{\sum_{k=1}^{10} \frac{\lambda_i(\boldsymbol{\beta}^{(t)})^k e^{-\lambda_i(\boldsymbol{\beta}^{(t)})}}{k!}}$$

which is a truncated Poisson distribution with rate $\lambda_i(\boldsymbol{\beta}^{(t)}) = \exp(\alpha_i + X_i \boldsymbol{\beta}^{(t)})$. Therefore, if

$$\tilde{y}_i(\boldsymbol{\beta}^{(t)}) = \sum_{j=1}^{10} j \frac{\frac{\lambda_i(\boldsymbol{\beta}^{(t)})^j e^{-\lambda_i(\boldsymbol{\beta}^{(t)})}}{j!}}{\sum_{k=1}^{10} \frac{\lambda_i(\boldsymbol{\beta}^{(t)})^k e^{-\lambda_i(\boldsymbol{\beta}^{(t)})}}{k!}},$$

$$\tilde{z}_i(\boldsymbol{\beta}^{(t)}) = \sum_{j=1}^{10} \log(j!) \frac{\frac{\lambda_i(\boldsymbol{\beta}^{(t)})^j e^{-\lambda_i(\boldsymbol{\beta}^{(t)})}}{j!}}{\sum_{k=1}^{10} \frac{\lambda_i(\boldsymbol{\beta}^{(t)})^k e^{-\lambda_i(\boldsymbol{\beta}^{(t)})}}{k!}},$$

then

$$\begin{aligned}Q(\boldsymbol{\beta}|\boldsymbol{\beta}^{(t)}) :&= E_{\mathbf{Y}_{missing}|\mathbf{C},\mathbf{Y}_{obs},\boldsymbol{\beta}^{(t)}} \log L(\boldsymbol{\beta}\,;\,\mathbf{C},\mathbf{Y}_{obs},\mathbf{Y}_{missing}) \\ &= \sum_{i\in\mathbf{W}} \left[\tilde{y}_i(\boldsymbol{\beta}^{(t)}) log(\lambda_i(\boldsymbol{\beta})) - \lambda_i(\boldsymbol{\beta}) - \tilde{z}_i(\boldsymbol{\beta}^{(t)})\right] + \\ &\quad \sum_{i\notin\mathbf{W}} \left[y_i \log(\lambda_i(\boldsymbol{\beta})) - \lambda_i(\boldsymbol{\beta}) - \log(y_i!)\right].\end{aligned}$$

Next, in the M step, the objective is to find the parameters that maximize $Q(\boldsymbol{\beta}|\boldsymbol{\beta}^{(t)})$ i.e. find $\boldsymbol{\beta}^{(t+1)} = arg\ max_{\boldsymbol{\beta}} Q(\boldsymbol{\beta}|\boldsymbol{\beta}^{(t)})$. Let

$$\begin{aligned}\tilde{Q}(\boldsymbol{\beta}|\boldsymbol{\beta}^{(t)}) = &\sum_{i\in\mathbf{W}} \left[\tilde{y}_i(\boldsymbol{\beta}^{(t)}) log(\lambda_i(\boldsymbol{\beta})) - \lambda_i(\boldsymbol{\beta}) - \log(\tilde{y}_i(\boldsymbol{\beta}^{(t)})!)\right] + \\ &\sum_{i\notin\mathbf{W}} \left[y_i \log(\lambda_i(\boldsymbol{\beta})) - \lambda_i(\boldsymbol{\beta}) - \log(y_i!)\right]\end{aligned}$$

Since $Q(\boldsymbol{\beta}|\boldsymbol{\beta}^{(t)})$ and $\tilde{Q}(\boldsymbol{\beta}|\boldsymbol{\beta}^{(t)})$ only differ by a constant as functions of $\boldsymbol{\beta}$, they have the same maximize. Notice that $\tilde{Q}(\boldsymbol{\beta}|\boldsymbol{\beta}^{(t)})$ is the log likelihood of a Poisson regression in which the outcomes are $(\tilde{y}_i(\boldsymbol{\beta}^{(t)}), i \in \mathbf{W})$ and $(y_i, i \notin \mathbf{W})$.

Therefore the M step can be done by fitting a Poisson regression with mean-imputed data for interval-censored outcomes, and this is equivalent to performing a Newton-Raphson method to get $\boldsymbol{\beta}$ in each step.
