# Supplementary material for: KGR-SKATER: Spatially clustered kernel graph regression for counting processes
Source: PLoS One. 2026 May 20;21(5):e0348787. doi: 10.1371/journal.pone.0348787 (PMC13189423; doi:10.1371/journal.pone.0348787)

S4 Appendix for KGR-SKATER: Spatially Clustered Kernel Graph Regression for Counting Processes

Jeffrey Wu[1,,¤*], Gareth W. Peters[1,¤,*], Alex Franks[1,¤,*],

1 Department of Statistics & Applied Probability, UCSB, Santa Barbara, California, USA

¤5607 South Hall Santa Barbara, CA 93106-2014, USA
* jeffreywu@pstat.ucsb.edu,garethpeters@pstat.ucsb.edu,afranks@pstat.ucsb.edu

S4: Silhouette plot to determine optimal number of clusters

This appendix has silhouette plots created using *fviznbclust()* to determine the optimal number of clusters

Fig S4.1. Silhouette plot of optimal number of clusters obtained via fviznbclust(). While 2 clusters has the largest average silhouette width, there is not a big drop off to 7 clusters so an analysis for 7 clusters was conducted in addition to 2.

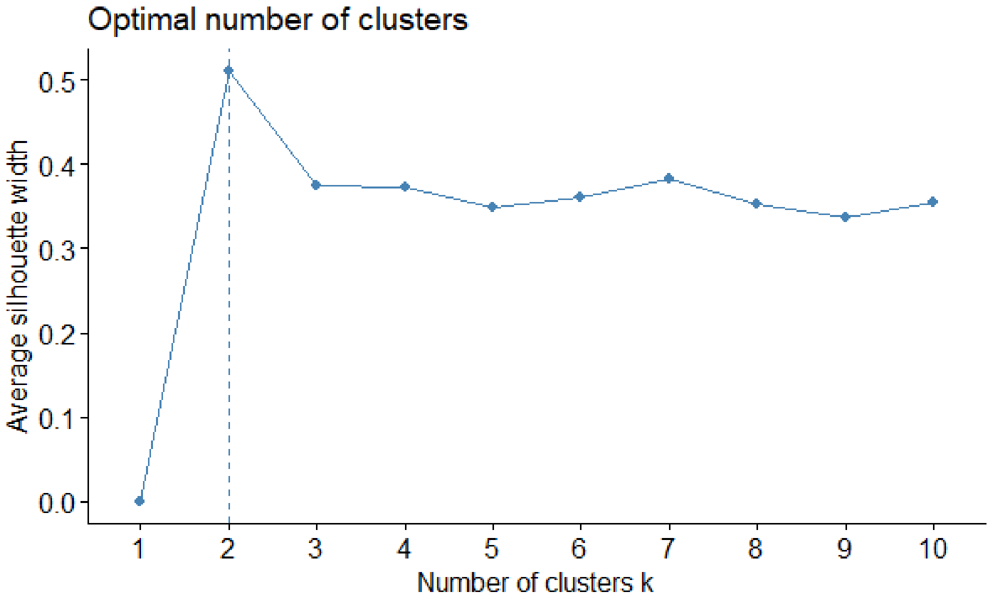

Supplement: S4 Appendix — (PDF) [file pone.0348787.s004.pdf]
