# Supplementary material for: KGR-SKATER: Spatially clustered kernel graph regression for counting processes
Source: PLoS One. 2026 May 20;21(5):e0348787. doi: 10.1371/journal.pone.0348787 (PMC13189423; doi:10.1371/journal.pone.0348787)
Supplement: S5 Appendix — (PDF) [file pone.0348787.s005.pdf]

S5 Appendix for KGR-SKATER: Spatially Clustered Kernel Graph Regression for Counting Processes

Jeffrey Wu[1,,¤*], Gareth W. Peters[1,¤,*], Alex Franks[1,¤,*],

1 Department of Statistics & Applied Probability, UCSB, Santa Barbara, California, USA

¤5607 South Hall Santa Barbara, CA 93106-2014, USA
* jeffreywu@pstat.ucsb.edu,garethpeters@pstat.ucsb.edu,afranks@pstat.ucsb.edu

S5: SKATER experiments

This appendix has results for some of the preliminary experiments performed with SKATER with respect to the application study. The purpose was to see if changing the inputs into the SKATER steps would lead to different cluster groupings (holding the constraint setting fixed).

Fig S5.1. Here are SKATER cluster groups (unconstrained) on different subsets of indices. County boundary shapefiles obtained from the US Census Bureau (`https://catalog.data.gov/dataset/tiger-line-shapefile-2016-state-california-current-place-state-based`). These are in the public domain. Maps were generated by the authors using R packages (*maps, sf, ggplot2*). The first row are cluster groups estimated on a single subindex variable (adjusted household income, % of individuals below the poverty line, and % of individuals working white collar jobs). The second row has groups of subindices: the first one is all of the subindices which make up the total SDI score, the second one is the two education subindices, and the last one is the three income subindices.

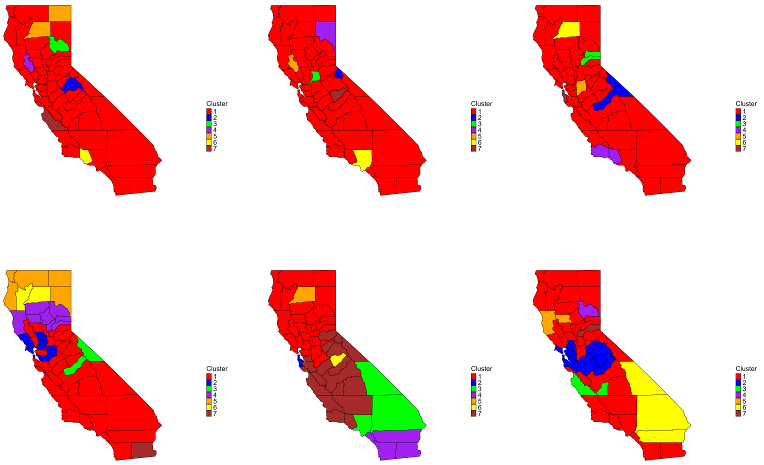

The same experiment with subindices is performed with the minimum population constraint.

Fig S5.2. Here are SKATER cluster groups (minimum population constraint) on different subsets of indices. County boundary shapefiles obtained from the US Census Bureau (`https://catalog.data.gov/dataset/tiger-line-shapefile-2016-state-california-current-place-state-based`). These are in the public domain. Maps were generated by the authors using R packages (*maps, sf, ggplot2*). The first row are cluster groups estimated on a single subindex variable (adjusted household income, % of individuals below the poverty line, and % of individuals working white collar jobs). The second row has groups of subindices: the first one is all of the subindices which make up the total SDI score, the second one is the two education subindices, and the last one is the three income subindices.

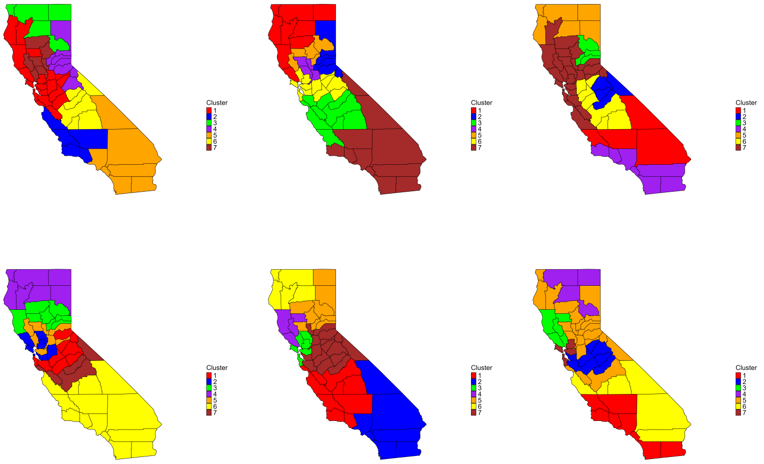

From the plots above, one can see that the SKATER cluster groupings are sensitive to the deprivation indices that are fed into the algorithm. Each of the plots is quite distinct, especially in S5.2. None of them really match the actual cluster grouping used in the actual application study, except maybe the cluster produced with the minimum population constraint on the three income subindices. At the end of the day, figuring out which counties should go into which spatial clusters was not particularly of interest in this application study, as long as the clusters generally made sense and were balanced. So, instead of only using a subset of the deprivation subindices, the SDI score itself was used because, by definition, it encapsulates the information from all of the subindices. It also seems more straightforward to calculate dissimilarity and prune a minimum spanning tree across one dimension as opposed to multiple.
