# Supplementary material for: KGR-SKATER: Spatially clustered kernel graph regression for counting processes
Source: PLoS One. 2026 May 20;21(5):e0348787. doi: 10.1371/journal.pone.0348787 (PMC13189423; doi:10.1371/journal.pone.0348787)
Supplement: S6 Appendix — (PDF) [file pone.0348787.s006.pdf]

S6 Appendix for KGR-SKATER: Spatially Clustered Kernel Graph Regression for Counting Processes

Jeffrey Wu[1,,¤*], Gareth W. Peters[1,¤,*], Alex Franks[1,¤,*],

1 Department of Statistics & Applied Probability, UCSB, Santa Barbara, California, USA

¤5607 South Hall Santa Barbara, CA 93106-2014, USA
* jeffreywu@pstat.ucsb.edu,garethpeters@pstat.ucsb.edu,afranks@pstat.ucsb.edu

S6: Evaluating different HUGE model selection criteria

This appendix has the results from some preliminary experiments with the options for the *huge()* function. There are several different model selection criteria (RIC, EBIC, and STARs) to select the best graph from a few estimates that HUGE provides. There is also an input λ which will be discussed at the end of this section.

To evaluate the differences between the model selection criteria mentioned above, several datasets of different dimensions were simulated with another function within the *huge* package, *huge.generator()*, which allows one to generate data multivariate normal data of different structures, such as banded or clustered. This function turned out to be useful for other simulation studies as well. The *huge.generator()* function not only outputs synthetic data, but also the covariance, precision, and/or adjacency matrices that were used to generate said data i.e. the true graph structure. So, under each simulated scenario, a synthetic dataset was fed into *huge* and the estimated graphs chosen by each model selection criteria were compared with a confusion matrix created by checking against the true graph structure.

S6.1 For 5 units with 10 data points each

Table S6.1. RIC, EBIC, STARS confusion matrices

Criterion	Predicted True	Predicted False
**RIC**		
**Actual True**	4	4
**Actual False**	8	9
**EBIC**		
**Actual True**	9	0
**Actual False**	8	8
**STARS**		
**Actual True**	9	0
**Actual False**	8	8

S6.2 For 5 units with 100 data points each

Table S6.2. RIC, EBIC, STARS confusion matrices

Criterion	Predicted True	Predicted False
**RIC**		
**Actual True**	8	0
**Actual False**	0	17
**EBIC**		
**Actual True**	8	0
**Actual False**	0	17
**STARS**		
**Actual True**	8	0
**Actual False**	0	17

S6.3 For 5 units with 1000 data points each

Table S6.3. RIC, EBIC, STARS confusion matrices

Criterion	Predicted True	Predicted False
**RIC**		
**Actual True**	8	0
**Actual False**	4	13
**EBIC**		
**Actual True**	8	0
**Actual False**	4	13
**STARS**		
**Actual True**	8	0
**Actual False**	4	13

S6.4 For 58 units with 200 data points

Table S6.4. RIC, EBIC, STARS confusion matrices

Criterion	Predicted True	Predicted False
**RIC**		
**Actual True**	0	142
**Actual False**	0	3052
**EBIC**		
**Actual True**	0	142
**Actual False**	0	3052
**STARS**		
**Actual True**	0	142
**Actual False**	0	3052

As one can see, there is very little difference between using RIC, EBIC, and STARS as the model selection criteria. Sometimes, the results of RIC diverge from those of EBIC and STARS like in Fig S6.1, which is the case most similar to the clustered SDI

data in the application study. Furthermore, not much background on RIC could be found in the literature review so there was some hesitation to rely on it as a criterion.

The big differentiator turns out to be the regularization parameter λ, input into the *huge* function. *huge* feeds λ in as a starting point for a sequence of decreasing values that control how sparse the graph to be estimated will be. When data of different sample sizes and dimensions were created while keeping the model selection criteria the same, it turns out that a $\lambda \geq 1$ always resulted in an estimated graph that was completely disconnected. Hence, λ was constrained to be less than or equal to 1, because it was established by calculating partial correlations using linear regression i.e., correlations between residuals, that there should be at least some edges when estimating a graph on deprivation score.
