# Supplementary material for: KGR-SKATER: Spatially clustered kernel graph regression for counting processes
Source: PLoS One. 2026 May 20;21(5):e0348787. doi: 10.1371/journal.pone.0348787 (PMC13189423; doi:10.1371/journal.pone.0348787)
Supplement: S7 Appendix — (PDF) [file pone.0348787.s007.pdf]

S7 Appendix for KGR-SKATER: Spatially Clustered Kernel Graph Regression for Counting Processes

Jeffrey Wu[1,,¤*], Gareth W. Peters[1,¤,*], Alex Franks[1,¤,*],

1 Department of Statistics & Applied Probability, UCSB, Santa Barbara, California, USA

¤5607 South Hall Santa Barbara, CA 93106-2014, USA
* jeffreywu@pstat.ucsb.edu,garethpeters@pstat.ucsb.edu,afranks@pstat.ucsb.edu

S7: Simulation study 1

This appendix outlines a simple simulation study conducted to ensure that the reference and proposed models introduced in the main paper could be implemented correctly in INLA.

S7.1 Data generating process

A time series of length 72 months was simulated from an LGCP using the *huge.generator()* and *mvrnorm()* functions in R. First, *huge.generator()* is used to generate data with a covariance structure exhibiting 50 clusters.

Fig S7.1. Simulated data with 50 clusters, and 72 observations per cluster. 50 clusters were used in this study instead of something more akin to the application study to make sure that INLA was still efficient when modeling higher dimensional data.

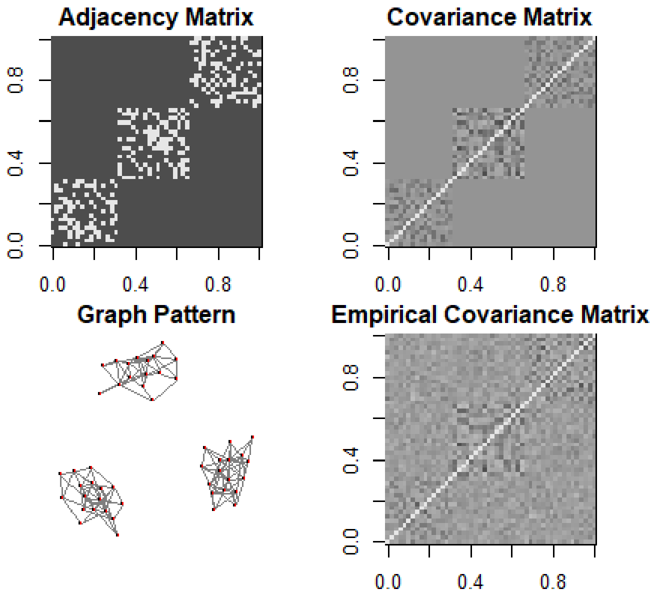

Using that data's covariance matrix and a mean of 5 for all clusters, an intensity $\boldsymbol{\Lambda}$ was simulated at all times and locations using *mvrnorm()* and then subsequently the outcome $y_{i,t}$ from a Poisson distribution with a seasonal pattern. So $y_{i,t} \sim Poisson(exp(sin(2\pi\frac{t}{12}) + \lambda_{i,t}))$. 1000 datasets were simulated this way to compare average RMSEs and parameter estimates.

S7.2 Results

The posterior predictive fits accuracy and parameter estimates for each model are shown in Figs S7.2 and S7.3. Each model had a fixed effect for the month but a different specification for the random effect i.e. covariance structure. For the Poisson GLMM, an *iid* covariance structure was used. For the BYM model, the adjacency matrix created by *huge.generator()* to generate the clustered structure was used for the graph specification. For the model option that will be used to implement KGR-SKATER models, *generic0*, the inverse of the sample covariance matrix created by *huge.generator()* was used as the fixed precision matrix. This seemed like a reasonable imitation of the KGR-SKATER methodology because in the application study, a fixed precision matrix obtained from covariate data will be used.

Fig S7.2. Boxplots of RMSE forecasting values for each model. Each boxplot represents the distribution of forecasting error at a given time point over 1000 simulations.

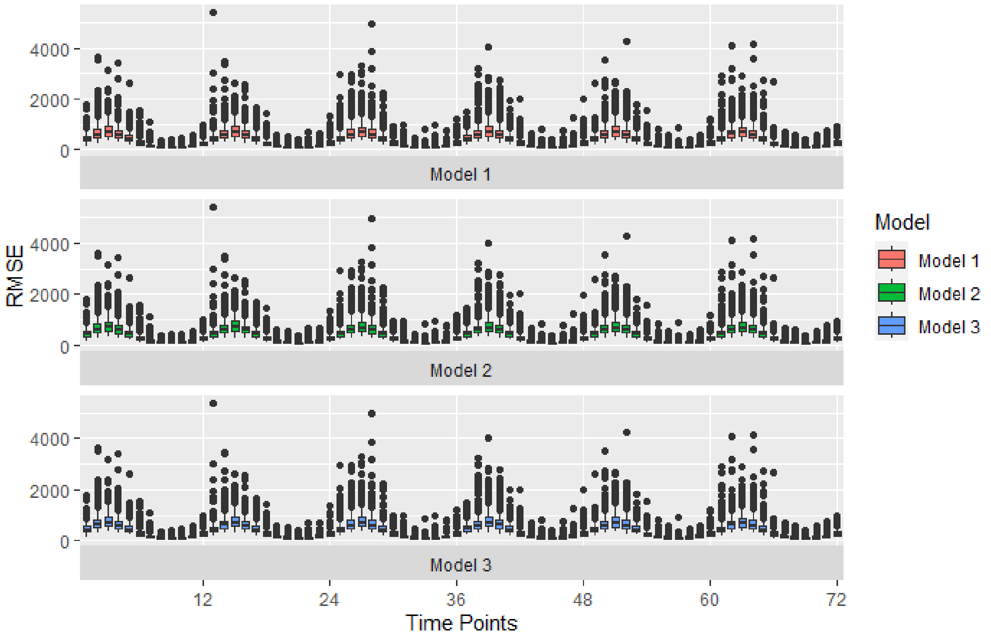

Fig S7.3. Density plots of estimated intensity by each model over 1000 simulations. The estimated intensity is virtually the same across the three models.

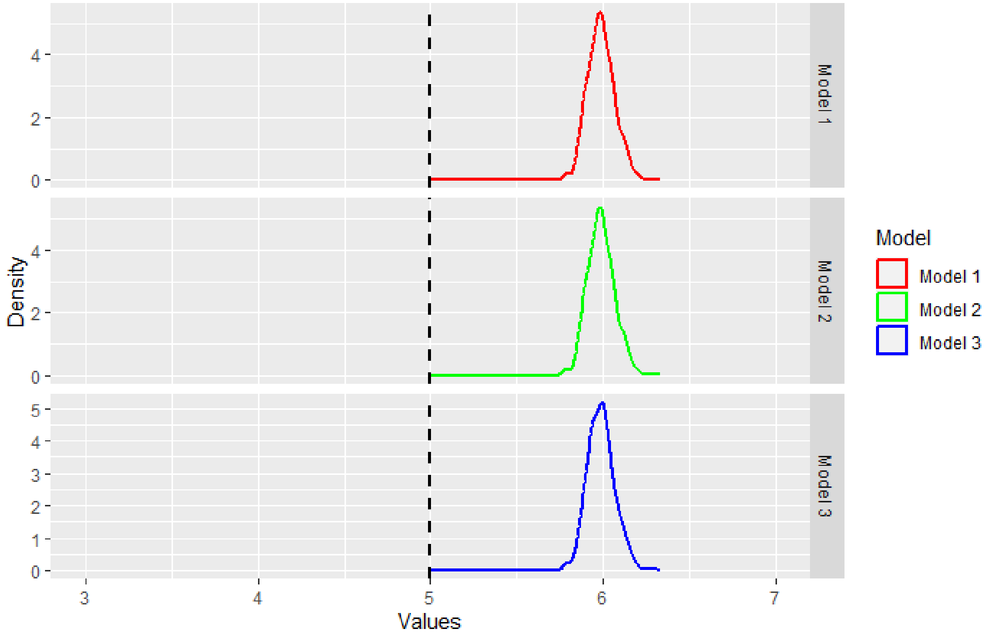

In conclusion, INLA models fit the data adequately and have very similar estimates. On average, model 1 estimated the intensity to be 5.981442, model 2 estimated the intensity to be 5.981478, and model 3 estimated the intensity to be 5.982072. INLA does seem to implement these models well; however, the default prior and hyperprior specifications do not appear to work very well. The credible bands for each model are too small, highlighting the need to perform hyperparameter learning before the model fitting step.
