# Supplementary material for: KGR-SKATER: Spatially clustered kernel graph regression for counting processes
Source: PLoS One. 2026 May 20;21(5):e0348787. doi: 10.1371/journal.pone.0348787 (PMC13189423; doi:10.1371/journal.pone.0348787)
Supplement: S8 Appendix — (PDF) [file pone.0348787.s008.pdf]

S8 Appendix for KGR-SKATER: Spatially Clustered Kernel Graph Regression for Counting Processes

Jeffrey Wu[1,,¤*], Gareth W. Peters[1,¤,*], Alex Franks[1,¤,*],

1 Department of Statistics & Applied Probability, UCSB, Santa Barbara, California, USA

¤5607 South Hall Santa Barbara, CA 93106-2014, USA
* jeffreywu@pstat.ucsb.edu,garethpeters@pstat.ucsb.edu,afranks@pstat.ucsb.edu

S8: Complete collection of SKATER and graph filter plots

This appendix presents the estimated spatial dependence structure for two and seven clusters estimated under every SKATER option outlined in the main paper.

As is illustrated below, the two cluster example is not particularly interesting. Two of the cluster groupings are the same, and the minimum population constraint clustering seems to just be splitting California into north and south. Furthermore, the same graph was estimated in each case (nodes are connected), so the graph filters are identical as well. Graph filters are calculated from the adjacency matrix estimated by HUGE. Thus, the same graph must produce the same graph filter. $\widetilde{\boldsymbol{L}}^2$ goes into the KGR-SKATER model's covariance matrix to induce smoothing between different spatial units.

Fig S8.1. Spatial dependence estimated for 2 clusters. County boundary shapefiles obtained from the US Census Bureau (`https://catalog.data.gov/dataset/tiger-line-shapefile-2016-state-california-current-place-state-based`). These are in the public domain. Maps were generated by the authors using R packages (*maps, sf, ggplot2*). The first row is obtained by running SKATER without constraints. The second row has the minimum population constraint. The third row has the minimum number of counties per cluster constraint.

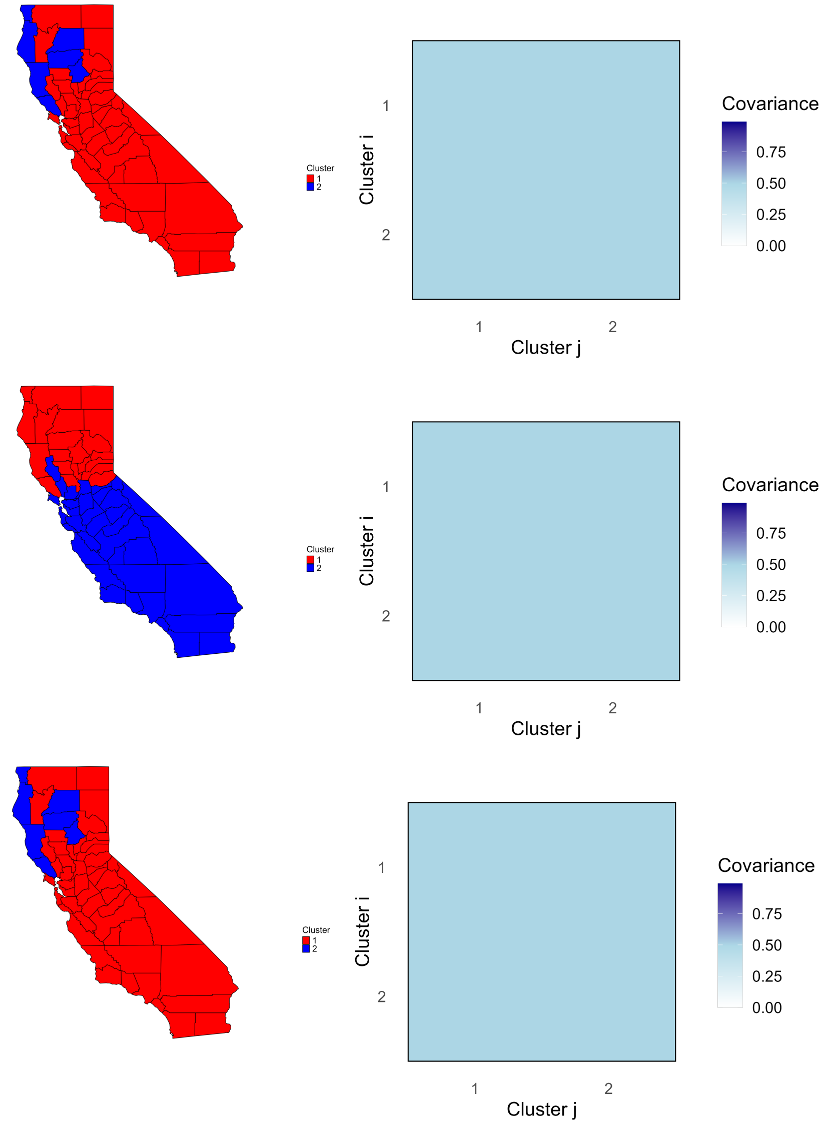

Fig S8.2. Spatial dependence estimated for 7 clusters. County boundary shapefiles obtained from the US Census Bureau (`https://catalog.data.gov/dataset/tiger-line-shapefile-2016-state-california-current-place-state-based`). These are in the public domain. Maps were generated by the authors using R packages (*maps, sf, ggplot2*). The first row is obtained by running SKATER without constraints. The second row has the minimum population constraint. The third row has the minimum number of counties per cluster constraint. Notice that the second two rows are the same, just with different colors.

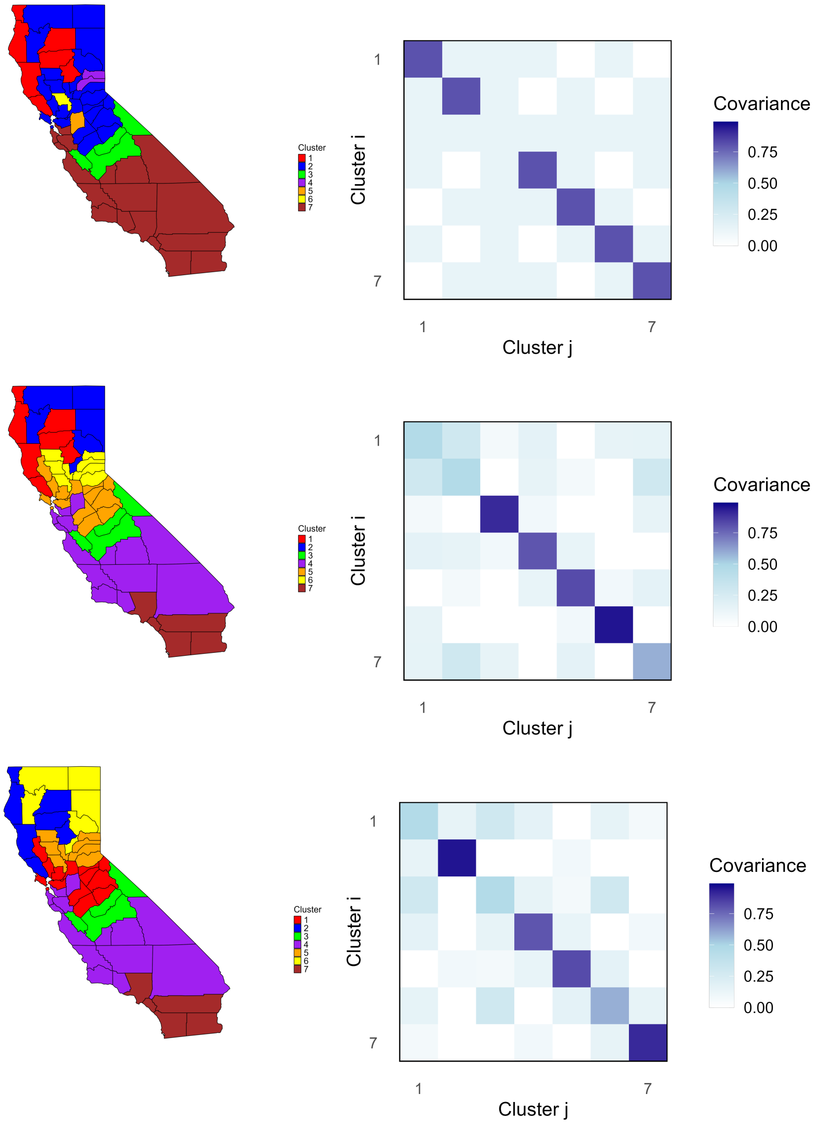

The graph filters for the seven cluster graphs are slightly different though. These slight differences in the entries of the graph filters, albeit small, can lead to differences in the estimation of model parameters which can be seen later on. Recall that the graph filter $\widetilde{\boldsymbol{L}}$ is important because it encodes the spatial dependence structure within the larger covariance/precision matrix of the LGCP models.
