# Supplementary material for: KGR-SKATER: Spatially clustered kernel graph regression for counting processes
Source: PLoS One. 2026 May 20;21(5):e0348787. doi: 10.1371/journal.pone.0348787 (PMC13189423; doi:10.1371/journal.pone.0348787)
Supplement: S9 Appendix — (PDF) [file pone.0348787.s009.pdf]

S9 Appendix for KGR-SKATER: Spatially Clustered Kernel Graph Regression for Counting Processes

Jeffrey Wu[1,,¤*], Gareth W. Peters[1,¤,*], Alex Franks[1,¤,*],

1 Department of Statistics & Applied Probability, UCSB, Santa Barbara, California, USA

¤5607 South Hall Santa Barbara, CA 93106-2014, USA
* jeffreywu@pstat.ucsb.edu,garethpeters@pstat.ucsb.edu,afranks@pstat.ucsb.edu

S9: Proposed KGR-SKATER model equations

This appendix contains the explicit modeling equations for each of the five KGR-SKATER models presented in the paper:

Proposed model 1:

$$
\begin{aligned}
&\widetilde{\boldsymbol{Y}}|\boldsymbol{\Lambda}, \boldsymbol{F}, \widetilde{\boldsymbol{S}} \sim Poisson(\boldsymbol{\Lambda}) \;, \\
&\quad \Lambda_{c,t}|\boldsymbol{F}, \widetilde{\boldsymbol{S}} = \exp\left(\sum_{c=1}^{C} \alpha_c \mathbb{I}\left[c \in \mathcal{C}_c\right] + \sum_{m=1}^{12} \beta_m \mathbb{I}\left[t \bmod m\right] + F_{c,t}\right) \;, \\
&\quad \boldsymbol{F}|\; \widetilde{\boldsymbol{S}} \sim \mathcal{GP}(\boldsymbol{0}, \boldsymbol{K} \otimes \widetilde{\boldsymbol{L}}^2) \;, \\
&\quad Cov(F_{c_1,t_1}, F_{c_2,t_2}|\widetilde{\boldsymbol{S}}) = \left[\boldsymbol{K}^{t-lp}\right]_{t_1,t_2} \left[\widetilde{\boldsymbol{L}}^2\right]_{c_1,c_2}
\end{aligned}
$$

Proposed model 2:

$$
\begin{aligned}
&\widetilde{\boldsymbol{Y}}|\boldsymbol{\Lambda}, \boldsymbol{F}, \widetilde{\boldsymbol{S}} \sim Poisson(\boldsymbol{\Lambda}) \;, \\
&\quad \Lambda_{c,t}|\boldsymbol{F}, \widetilde{\boldsymbol{S}} = \exp\left(\sum_{c=1}^{C} \alpha_c \mathbb{I}\left[c \in \mathcal{C}_c\right] + \sum_{m=1}^{12} \beta_m \mathbb{I}\left[t \bmod m\right] + F_{c,t}\right) \;, \\
&\quad \boldsymbol{F}|\; \widetilde{\boldsymbol{S}} \sim \mathcal{GP}(\boldsymbol{0}, \boldsymbol{K} \otimes \widetilde{\boldsymbol{L}}^2) \;, \\
&\quad Cov(F_{c_1,t_1}, F_{c_2,t_2}|\widetilde{\boldsymbol{S}}) = \left[\boldsymbol{K}^{x-lp}\right]_{t_1,t_2} \left[\widetilde{\boldsymbol{L}}^2\right]_{c_1,c_2}
\end{aligned}
$$

Proposed model 3:

$$
\begin{aligned}
&\widetilde{\boldsymbol{Y}}|\boldsymbol{\Lambda}, \boldsymbol{F}, \widetilde{\boldsymbol{S}} \sim Poisson(\boldsymbol{\Lambda}) \;, \\
&\quad \Lambda_{c,t}|\boldsymbol{F}, \widetilde{\boldsymbol{S}} = \exp\left(\sum_{c=1}^{C} \alpha_c \mathbb{I}\left[c \in \mathcal{C}_c\right] + \sum_{m=1}^{12} \beta_m \mathbb{I}\left[t \bmod m\right] + F_{c,t}\right) \;, \\
&\quad \boldsymbol{F}|\; \widetilde{\boldsymbol{S}} \sim \mathcal{GP}(\boldsymbol{0}, \boldsymbol{K} \otimes \widetilde{\boldsymbol{L}}^2) \;, \\
&\quad Cov(F_{c_1,t_1}, F_{c_2,t_2}|\widetilde{\boldsymbol{S}}) = \left[\boldsymbol{K}^{t-lp}\right]_{t_1,t_2} \left[\boldsymbol{K}^{x-lp}\right]_{t_1,t_2} \left[\widetilde{\boldsymbol{L}}^2\right]_{c_1,c_2}
\end{aligned}
$$

Proposed model 4: 7

$$\begin{aligned}
&\widetilde{\boldsymbol{Y}}|\boldsymbol{\Lambda},\boldsymbol{F},\widetilde{\boldsymbol{S}} \sim Poisson(\boldsymbol{\Lambda}) \ , \\
&\quad \Lambda_{c,t}|\boldsymbol{F},\widetilde{\boldsymbol{S}} = \exp\left(\sum_{c=1}^{C}\alpha_c \mathbb{I}\left[c \in \mathcal{C}_c\right] + \sum_{m=1}^{12}\beta_m \mathbb{I}\left[t \bmod m\right] + F_{c,t}\right) \ , \\
&\quad \boldsymbol{F}|\ \widetilde{\boldsymbol{S}} \sim \mathcal{GP}(\boldsymbol{0},\boldsymbol{K}\otimes\widetilde{\boldsymbol{L}}^2) \ , \\
&\quad Cov(F_{c_1,t_1},F_{c_2,t_2}|\widetilde{\boldsymbol{S}}) = \frac{\left(\left[\boldsymbol{K}^{t-lp}\right]_{t_1,t_2} + \left[\boldsymbol{K}^{x-lp}\right]_{t_1,t_2}\right)}{2}\left[\widetilde{\boldsymbol{L}}^2\right]_{c_1,c_2}
\end{aligned}$$

Proposed model 5: 8

$$\begin{aligned}
&\widetilde{\boldsymbol{Y}}|\boldsymbol{\Lambda},\boldsymbol{F},\widetilde{\boldsymbol{S}} \sim Poisson(\boldsymbol{\Lambda}) \ , \\
&\quad \Lambda_{c,t}|\boldsymbol{F},\widetilde{\boldsymbol{S}} = \exp\left(\sum_{c=1}^{C}\alpha_c \mathbb{I}\left[c \in \mathcal{C}_c\right] + \sum_{m=1}^{12}\beta_m \mathbb{I}\left[t \bmod m\right] + F_{c,t}\right) \ , \\
&\quad \boldsymbol{F}|\ \widetilde{\boldsymbol{S}} \sim \mathcal{GP}(\boldsymbol{0},\boldsymbol{K}\otimes\widetilde{\boldsymbol{L}}^2) \ , \\
&\quad Cov(F_{c_1,t_1},F_{c_2,t_2}|\widetilde{\boldsymbol{S}}) = \frac{\left(\left[\boldsymbol{K}^{bp-lp}\right]_{t_1,t_2} + \left[\boldsymbol{K}^{dl-lp}\right]_{t_1,t_2} + \left[\boldsymbol{K}^{idl-lp}\right]_{t_1,t_2}\right)}{3}\left[\widetilde{\boldsymbol{L}}^2\right]_{c_1,c_2}
\end{aligned}$$
