# Supplementary material for: KGR-SKATER: Spatially clustered kernel graph regression for counting processes
Source: PLoS One. 2026 May 20;21(5):e0348787. doi: 10.1371/journal.pone.0348787 (PMC13189423; doi:10.1371/journal.pone.0348787)
Supplement: S10 Appendix — (PDF) [file pone.0348787.s010.pdf]

S10 Appendix for KGR-SKATER: Spatially Clustered Kernel Graph Regression for Counting Processes

Jeffrey Wu[1,,¤*], Gareth W. Peters[1,¤,*], Alex Franks[1,¤,*],

1 Department of Statistics & Applied Probability, UCSB, Santa Barbara, California, USA

¤5607 South Hall Santa Barbara, CA 93106-2014, USA
* jeffreywu@pstat.ucsb.edu,garethpeters@pstat.ucsb.edu,afranks@pstat.ucsb.edu

S10: Model comparison criterion for application study

The Bayesian model selection criteria DIC and WAIC are useful for comparing hierarchical models.

$$\begin{aligned} &\text{DIC} = \bar{D} + p_D \ , \\ &\text{where } \bar{D} = E_{\theta|y}[D(\theta)] \ , \\ &\qquad p_D = E_{\theta|y}[D(\theta)] - D(E_{\theta|y}[\theta]) = \bar{D} - D(\bar{\theta}) \end{aligned}$$

where $D(\theta) = -2log(p(y|\theta))$, the first term is the posterior mean deviance i.e., a measure of fit, and the second term is the effective number of parameters i.e. a measure of model complexity.

$$\begin{aligned} &\text{WAIC} = T_n + \frac{V_n}{n} \ , \\ &\text{where } T_n = -\frac{1}{n}\sum_{i=1}^{n} logp^*(Y_i|w) \ , \\ &\qquad V_n = \sum_{i=1}^{n}\{E_w[(logp(Y_i|w))^2] - E_w[logp(Y_i|w)]^2\} \end{aligned}$$

where T_n is the log loss function and w represents the parameters of the model.

Based on these criteria, the best reference and proposed models are identified and carried into out of sample fitting. DIC and WAIC are similar metrics, in both cases, the smaller the value, the better the model. The main difference is that WAIC uses the variance instead of the average in log-likelihood over the posterior distribution as a measure of flexibility [1]. This results in harsher penalties when variances are high which may not be accounted by DIC [1].

Table S10.1. DIC and WAIC values for each model.

Reference Models		Proposed Models				
#1	#2	#1	#2	#3	#4	#5
**DIC for 2 clusters**						
834.708	**834.708**	991.672	994.237	995.189	992.573	994.487
**DIC for 7 clusters**						
2869.994	2869.994	2840.376	2843.095	2842.394	**2834.012**	2839.578
**WAIC for 2 clusters**						
1881.963	1881.963	**977.525**	980.437	981.191	978.203	980.494
**WAIC for 7 clusters**						
4065.576	4065.576	2818.437	2821.594	2820.723	**2808.770**	2815.812

One of the proposed models is preferred in three out of the four settings. Reference model 2 is preferred by DIC for two clusters. These results were obtained using SKATER's minimum population constraint.

Notice that with respect to WAIC, the reference models are apparently much less optimal compared to the proposed models. This is not the case however for DIC, where reference model 2 is actually preferred for two clusters. It is hard to say much about why there is this dissonance, especially non-asymptotically, beyond the fact that the two criteria have slightly different penalty terms.

Since reference model 3 specifies no spatial dependence between clusters, a separate model is fit for each cluster. This results in separate DIC and WAIC values for each cluster. Hence, for reference model 3's entries in the table, the average of each cluster's DIC and WAIC was calculated. This DIC and WAIC values for two and seven clusters for this model are: $596.273, 371.816, 580.352, 364.606$.

References

1. Evans NJ. Assessing the practical differences between model selection methods in inferences about choice response time tasks. 2019;26(4):1070-98. Available from: https://doi.org/10.3758/s13423-018-01563-9. doi:10.3758/s13423-018-01563-9.
