# Supplementary material for: KGR-SKATER: Spatially clustered kernel graph regression for counting processes
Source: PLoS One. 2026 May 20;21(5):e0348787. doi: 10.1371/journal.pone.0348787 (PMC13189423; doi:10.1371/journal.pone.0348787)

S11 Appendix for KGR-SKATER: Spatially Clustered Kernel Graph Regression for Counting Processes

Jeffrey Wu[1,,¤*], Gareth W. Peters[1,¤,*], Alex Franks[1,¤,*],

1 Department of Statistics & Applied Probability, UCSB, Santa Barbara, California, USA

¤5607 South Hall Santa Barbara, CA 93106-2014, USA
* jeffreywu@pstat.ucsb.edu,garethpeters@pstat.ucsb.edu,afranks@pstat.ucsb.edu

S11: Heatmaps of precision matrix of underlying GP of LGCP models

This appendix includes heatmaps of the precision matrices of the underlying GPs of each of the models that take the form of LGCPs i.e. reference model 3 and all of the proposed models. The precision matrix was plotted instead of the covariance matrix because of the convenient, interpretable property of 0 entries within the precision matrix of a GP.

Fig S11.1. Heatmaps of precision matrix of underlying GP of LGCP models. Row 1: $\mathcal{M}_3^R$ and $\mathcal{M}_1$. Row 2: $\mathcal{M}_2$ and $\mathcal{M}_3$. Row 3: $\mathcal{M}_4$ and $\mathcal{M}_5$. Most of the precision matrices are sparse, meaning that most spatial unit/time point combination pairs ij are conditionally independent (i.e., entry i is not correlated with entry j). Notable exceptions are the matrices for reference model 3 (which makes sense because it is a locally periodic time kernel) and $\mathcal{M}_1$.

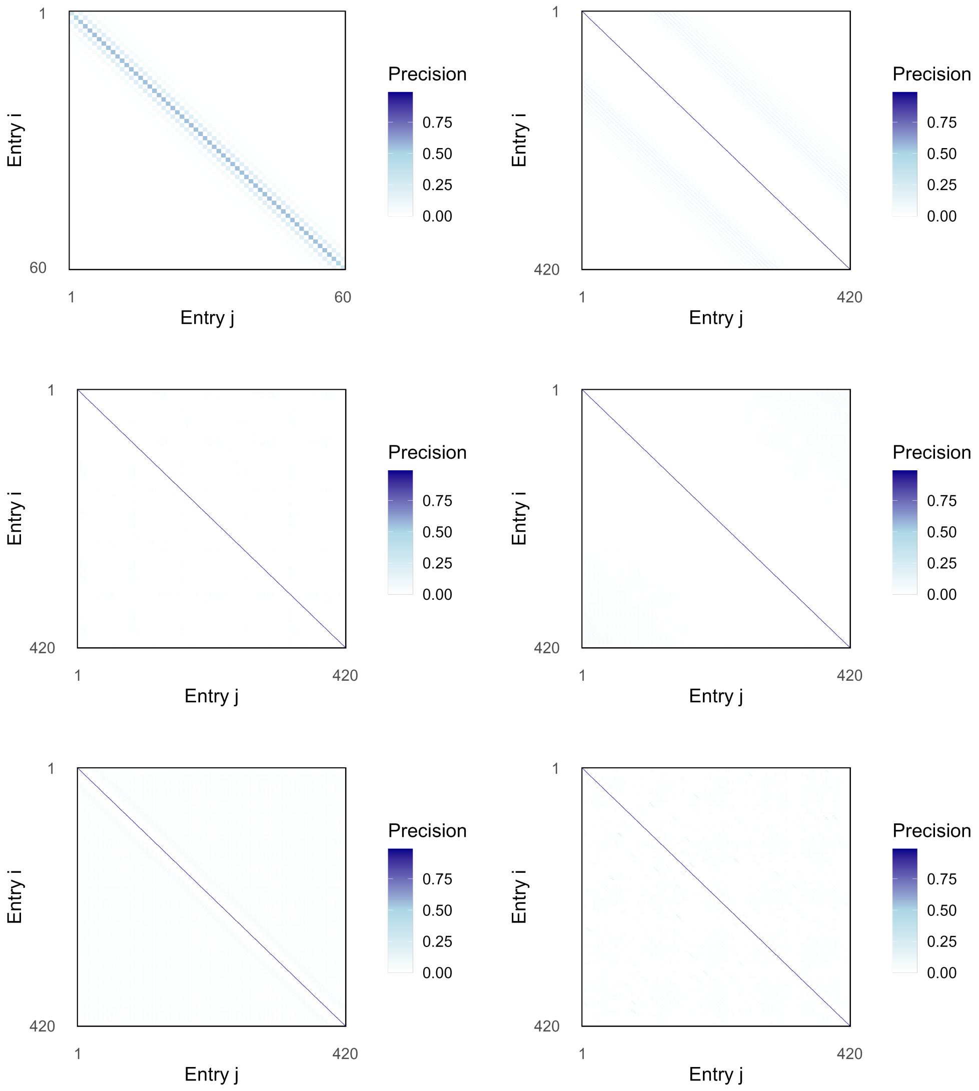

Supplement: S11 Appendix — (PDF) [file pone.0348787.s011.pdf]
