# Supplementary material for: KGR-SKATER: Spatially clustered kernel graph regression for counting processes
Source: PLoS One. 2026 May 20;21(5):e0348787. doi: 10.1371/journal.pone.0348787 (PMC13189423; doi:10.1371/journal.pone.0348787)
Supplement: S12 Appendix — (PDF) [file pone.0348787.s012.pdf]

# S12 Appendix for KGR-SKATER: Spatially Clustered Kernel Graph Regression for Counting Processes

Jeffrey Wu[1,,¤*], Gareth W. Peters[1,¤,*], Alex Franks[1,¤,*],

**1** Department of Statistics & Applied Probability, UCSB, Santa Barbara, California, USA

¤5607 South Hall Santa Barbara, CA 93106-2014, USA
* jeffreywu@pstat.ucsb.edu,garethpeters@pstat.ucsb.edu,afranks@pstat.ucsb.edu

## S12: In sample RMSPE table for reference and proposed models

This appendix contains the in sample RMSPEs for the reference and proposed models fit in the application study of the main paper.

**Table S12.1. Table of in-sample RMSPE for each model.**

| | Reference Models | | Proposed Models | | | | |
|---|---|---|---|---|---|---|---|
| | #2 | #3 | #1 | #2 | #3 | #4 | #5 |
| **2 clusters** | | | | | | | |
| Cluster 1 | 0.2294 | **0.2115** | 0.2281 | 0.2273 | 0.2268 | 0.2276 | 0.2772 |
| Cluster 2 | **0.2294** | 0.2433 | 0.2435 | 0.2435 | 0.2435 | 0.2345 | 0.2435 |
| **7 clusters** | | | | | | | |
| Cluster 1 | 0.2294 | **0.1870** | 0.2297 | 0.2290 | 0.2284 | 0.2275 | 0.2271 |
| Cluster 2 | 0.2294 | **0.1863** | 0.2310 | 0.2303 | 0.2303 | 0.2309 | 0.2295 |
| Cluster 3 | **0.2294** | 0.2469 | 0.2480 | 0.2479 | 0.2478 | 0.2485 | 0.2483 |
| Cluster 4 | **0.2294** | 0.2675 | 0.2568 | 0.2574 | 0.2582 | 0.2605 | 0.2599 |
| Cluster 5 | 0.2294 | **0.2291** | 0.2362 | 0.2356 | 0.2355 | 0.2348 | 0.2343 |
| Cluster 6 | 0.2296 | **0.0012** | 0.2227 | 0.2218 | 0.2204 | 0.2161 | 0.2185 |
| Cluster 7 | **0.2294** | 0.2316 | 0.2350 | 0.2349 | 0.2348 | 0.2344 | 0.2345 |

Notice that the reference models and the proposed models produce similar in-sample fits even though the reference models outperform the proposed models (in terms of accuracy) for each cluster. Reference models 2 and 3 are remarkably consistent. These results were obtained using SKATER's minimum population constraint.

While the in sample RMSPEs is slightly better for the reference models than the proposed models, as shown in the main paper, the proposed models have better uncertainty quantification. This is likely due to the more rigorous specification of the spatiotemporal dependence structure in the KGR-SKATER fitting procedure.
