# Supplementary material for: KGR-SKATER: Spatially clustered kernel graph regression for counting processes
Source: PLoS One. 2026 May 20;21(5):e0348787. doi: 10.1371/journal.pone.0348787 (PMC13189423; doi:10.1371/journal.pone.0348787)

S13 Appendix for KGR-SKATER: Spatially Clustered Kernel Graph Regression for Counting Processes

Jeffrey Wu[1,¤,*], Gareth W. Peters[1,¤,*], Alex Franks[1,¤,*],

1 Department of Statistics & Applied Probability, UCSB, Santa Barbara, California, USA

¤5607 South Hall Santa Barbara, CA 93106-2014, USA
* jeffreywu@pstat.ucsb.edu,garethpeters@pstat.ucsb.edu,afranks@pstat.ucsb.edu

S13: Simulation study 2

It is a bit unfortunate, because of the predictable nature of the application study's time series, that the proposed model form's utility could not be demonstrated in situations where a time series may not have such a consistent period or amplitude as in the application study. For this reason, this appendix includes a simulation study that is conducted with a doubly stochastic Poisson process with a less consistent period and amplitude over a longer period of time (25 years or 300 months).

To create this data, 5 clusters with the following graph structure were created to simulate some spatial dependence structure. This graph structure was then used to calculate a corresponding graph filter.

Fig S13.1. Simulated spatial dependence represented by a graph. This sparse graph structure was created by design to see how an unusual dependence structure, e.g., a completely disconnected node, might affect modeling performance.

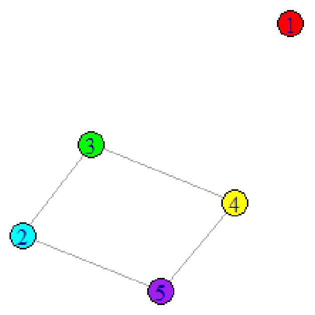

Next, a temporal dependence structure was generated based on the following temporal kernel which is the product of a RBF kernel and two periodic kernels, one at

six months and one at twelve months.

$$K(t_1, t_2; \sigma^2, \rho) = \sigma^2 exp(-\frac{2sin^2(\pi|t_1 - t_2|/12)}{\rho})exp(-\frac{2sin^2(\pi|t_1 - t_2|/6)}{\rho})exp(-\frac{\sum_{i=1}^{d}(t_1 - t_2)^2}{2\rho})$$

Then the Kronecker product is taken between the graph filter squared and this temporal kernel to get the synthetic covariance matrix of the DGP's underlying intensity function, which is assumed to be a Gaussian Process, like in the models that will be used. To ensure that one component of the covariance matrix is not dominating the other, a weight was applied based on the largest eigenvalue of each component, i.e.,

$$\Sigma = w\mathbf{K} \otimes (1 - w)\mathbf{H}^2 \,,$$
$$w = \frac{\lambda_{max}(\mathbf{K})^{-1}}{\lambda_{max}(\mathbf{K})^{-1} + \lambda_{max}(\mathbf{H}^2)^{-1}}$$

In order for the means of the underlying Gaussian Process to have some periodicity within a given year but vary completely between clusters and years, the following mean function was used:

$$m_t = A_t sin(\frac{2\pi}{12}t) + B_t sin(\frac{2\pi}{6}t) \,,$$
$$A_t, B_t \sim N(0, 1)$$

Using the Gaussian Process defined above ($\mathbf{F} \sim \mathcal{GP}(\mathbf{m}, \mathbf{\Sigma})$), which is of the dimension $5 * 300 = 1500$, a stochastic process was generated for the underlying intensity of each cluster's Poisson process. To emulate the fact that in the application study, some clusters were much larger than others, intercepts of different magnitudes were added to each cluster when converting the intensities into the mean intensities, i.e.,

$$\mathbf{\Lambda}_{c,t} = exp(\beta_c + \mathbf{F}_{c,t})$$

There is one last step of generating counts from the simulated mean intensities, but this was skipped because INLA cannot predict counts, only the corresponding average intensities. Therefore, this simulation study focuses on modeling $\Lambda_{c,t}$. The average intensities simulated for each cluster are displayed below:

Fig S13.2. Time series plots of simulated mean intensities for five different clusters, all of different scales. Notice the irregular periods and amplitudes of each time series.

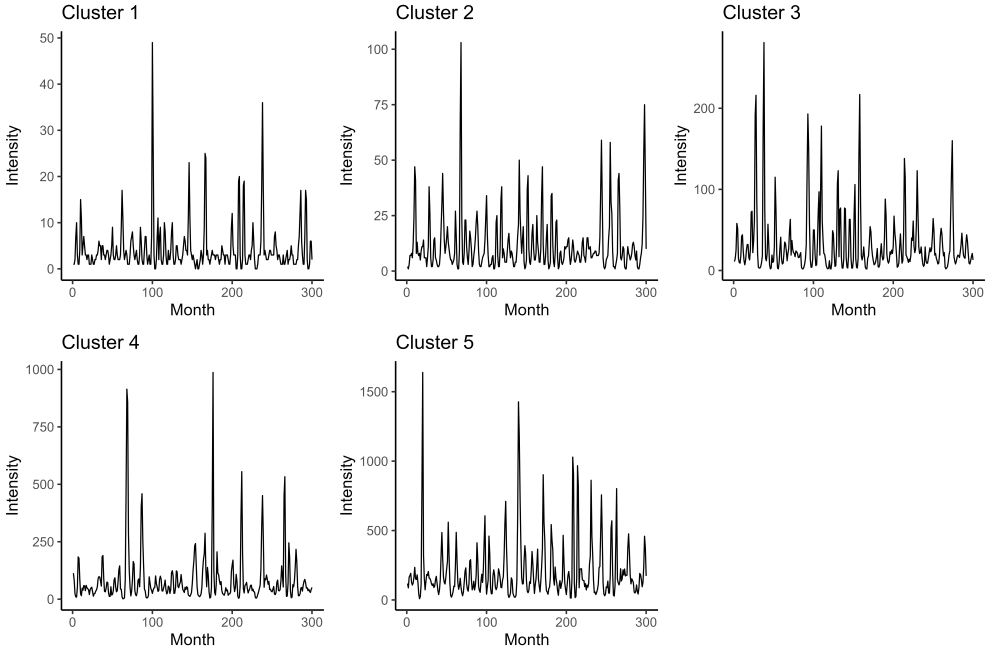

The posterior predictive plots below show the posterior predictive forecasts for a one month ahead forecast with a sliding in sample window with an origin at month 240. Clearly, the complex dependence structure encoded in the KGR model allows for more variation in predictions of the intensity. As stated before, this leads to better performance for messier time series.

Fig S13.3. INLA's posterior predictive fits for BYM model. The same pattern is being used over and over again in each cluster, but it is reasonably accurate. However, the credible interval bands are basically nonexistent.

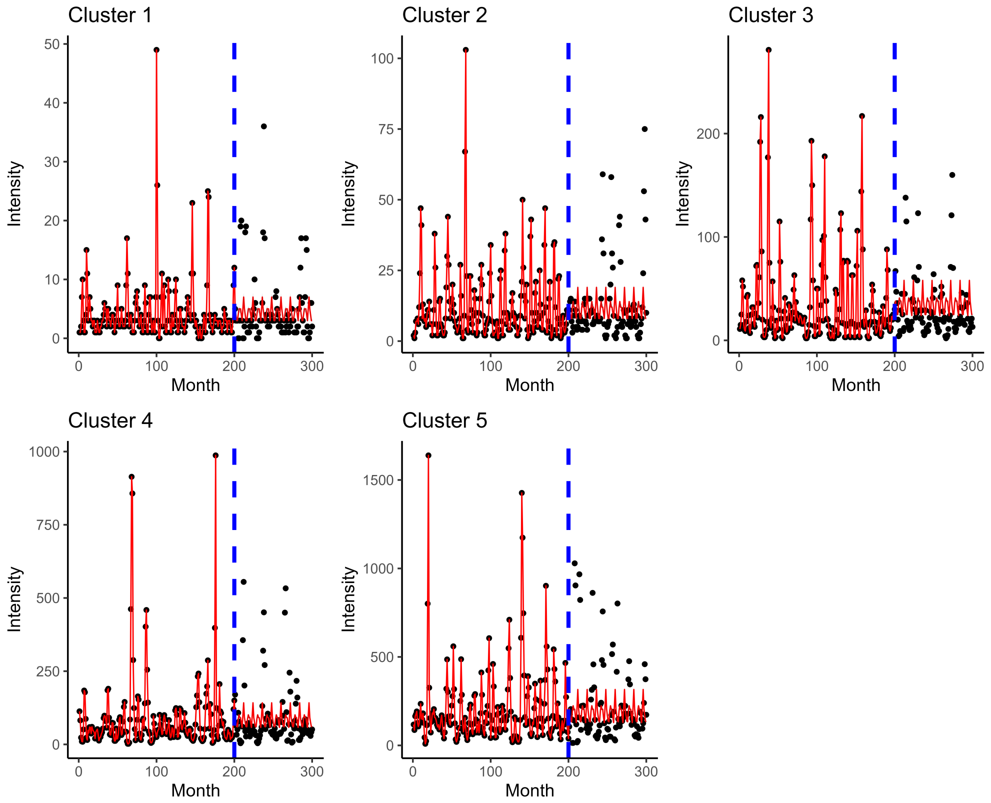

Fig S13.4. INLA's posterior predictive fits for KGR-SKATER model. With the exception of Cluster 1, the KGR-SKATER model seems to fit the synthetic data better, particularly when it comes to uncertainty quantification. The credible interval bands are probably a bit too wide if anything.

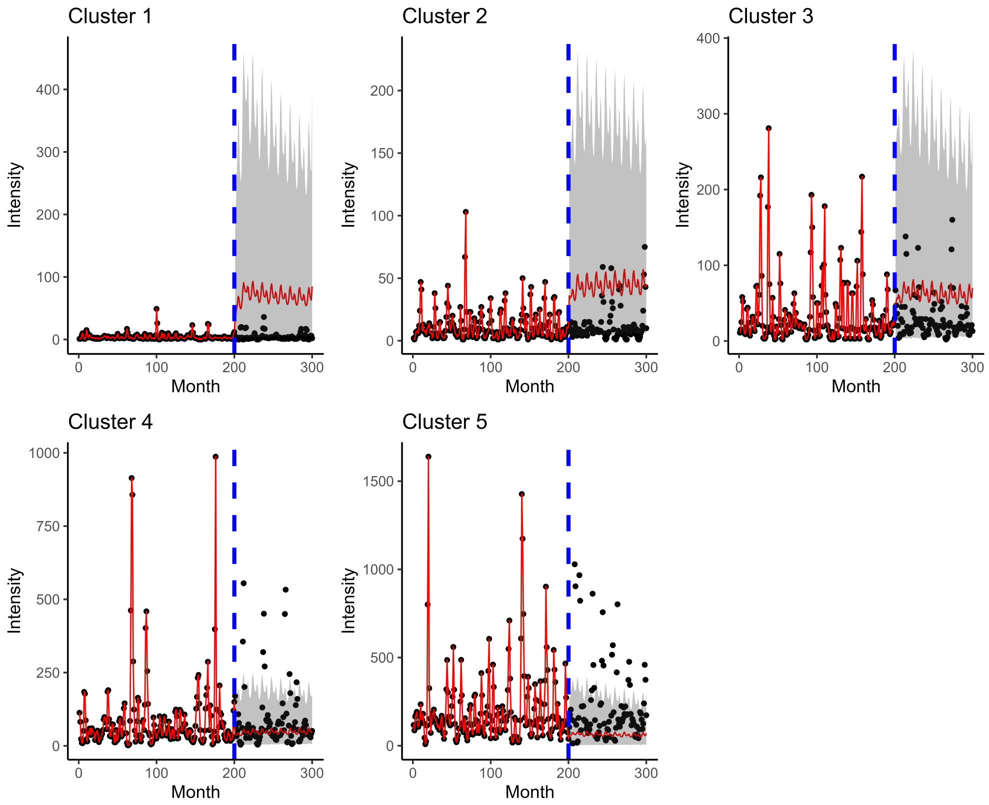

Supplement: S13 Appendix — (PDF) [file pone.0348787.s013.pdf]
