# Supplementary material for: KGR-SKATER: Spatially clustered kernel graph regression for counting processes
Source: PLoS One. 2026 May 20;21(5):e0348787. doi: 10.1371/journal.pone.0348787 (PMC13189423; doi:10.1371/journal.pone.0348787)

S14 Appendix for KGR-SKATER: Spatially Clustered Kernel Graph Regression for Counting Processes

Jeffrey Wu[1,,¤*], Gareth W. Peters[1,¤,*], Alex Franks[1,¤,*],

1 Department of Statistics & Applied Probability, UCSB, Santa Barbara, California, USA

¤5607 South Hall Santa Barbara, CA 93106-2014, USA
* jeffreywu@pstat.ucsb.edu,garethpeters@pstat.ucsb.edu,afranks@pstat.ucsb.edu

S14: Posterior predictive plots for other reference and proposed models

This appendix contains the out of sample posterior predictive plots for all of the other models presented in the paper besides $\mathcal{M}_2^R$ and $\mathcal{M}_4$:

Fig S14.1. Posterior predictive plots for reference model 1 (not included in results sections). Most of these models' posterior predictive estimates are quite similar to each other, with the exception of reference model 3.

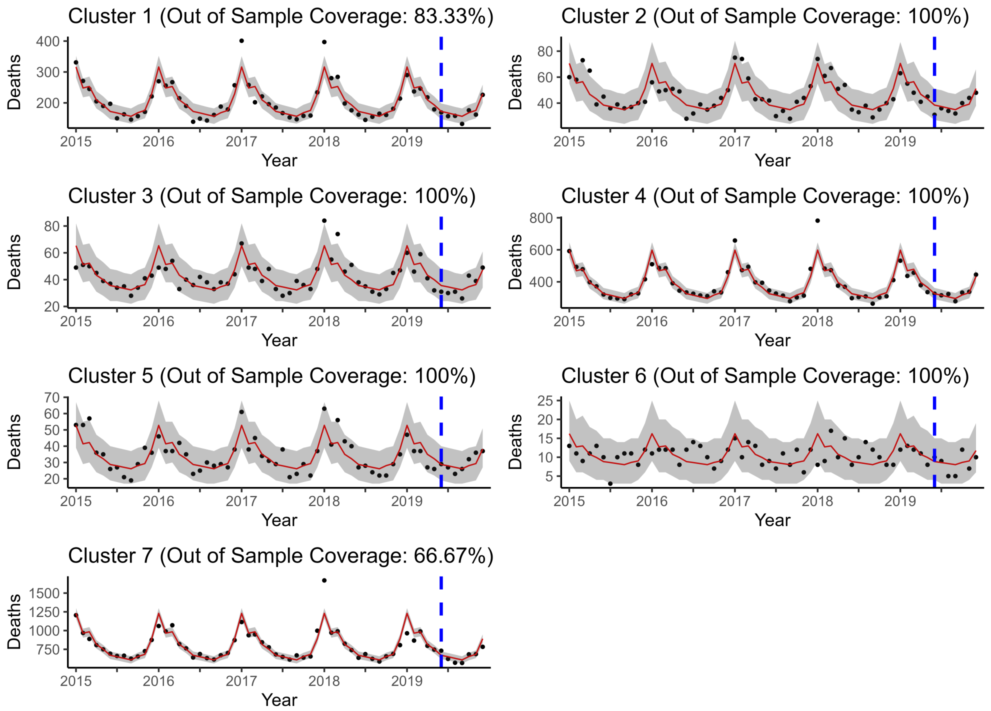

Fig S14.2. Posterior predictive plots for reference model 3 (not included in results sections). Most of these models' posterior predictive estimates are quite similar to each other, with the exception of reference model 3.

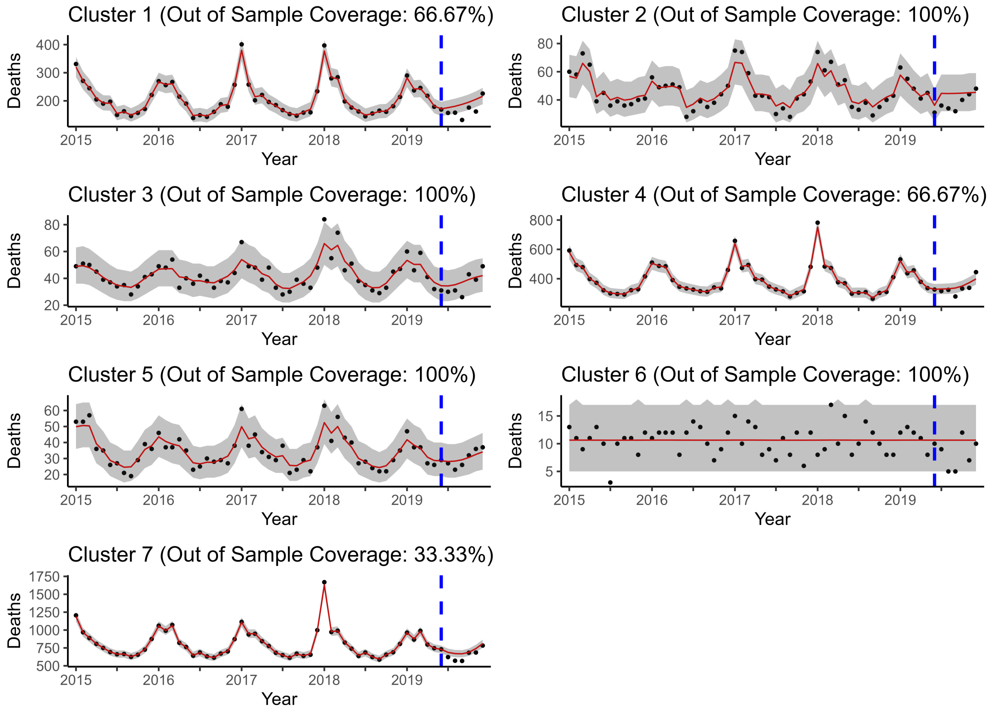

Reference model 3, which recall is an LGCP with a simple time kernel as its underlying covariance matrix, is a bit of a curious case in the application study because it performs very well for the most part. But in some instances like in cluster 6, this model generates a horizontal line as its prediction. It may be because the hyperparameter set chosen via grid search was incorrect and hence, the estimate of variation in the time series did not converge.

Fig S14.3. Posterior predictive plots for $\mathcal{M}_1$ (not included in results sections). Most of these models' posterior predictive estimates are quite similar to each other, with the exception of reference model 3.

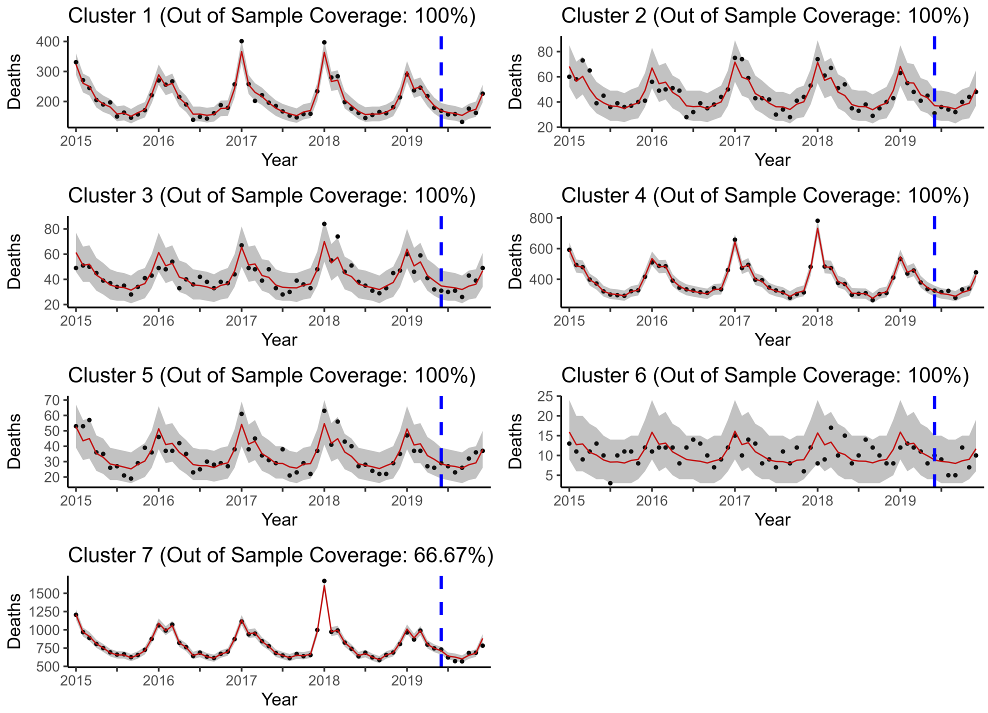

Fig S14.4. Posterior predictive plots for $\mathcal{M}_2$ (not included in results sections). Most of these models' posterior predictive estimates are quite similar to each other, with the exception of reference model 3.

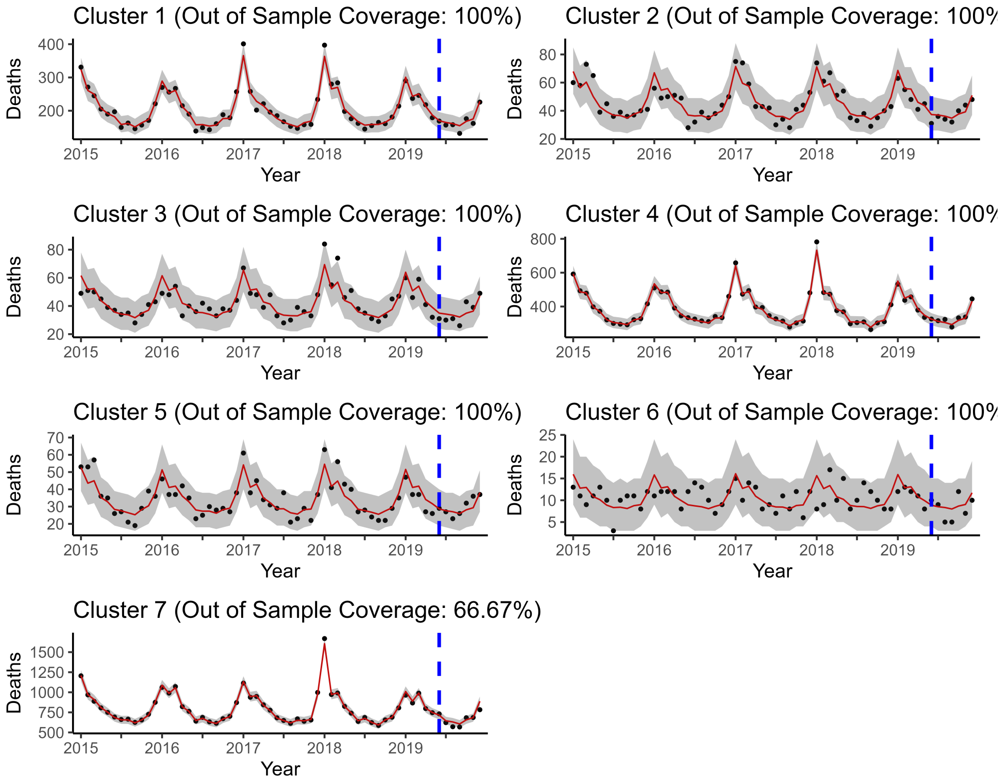

Fig S14.5. Posterior predictive plots for $\mathcal{M}_3$ (not included in results sections). Most of these models' posterior predictive estimates are quite similar to each other, with the exception of reference model 3.

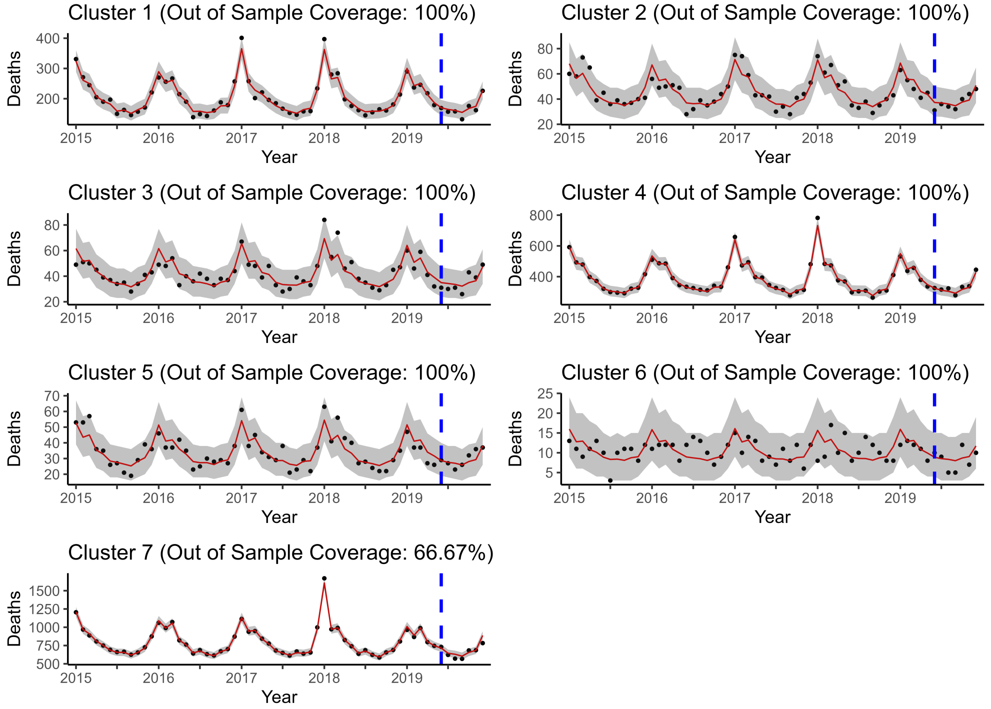

Fig S14.6. Posterior predictive plots for $\mathcal{M}_5$ (not included in results sections). Most of these models' posterior predictive estimates are quite similar to each other, with the exception of reference model 3.

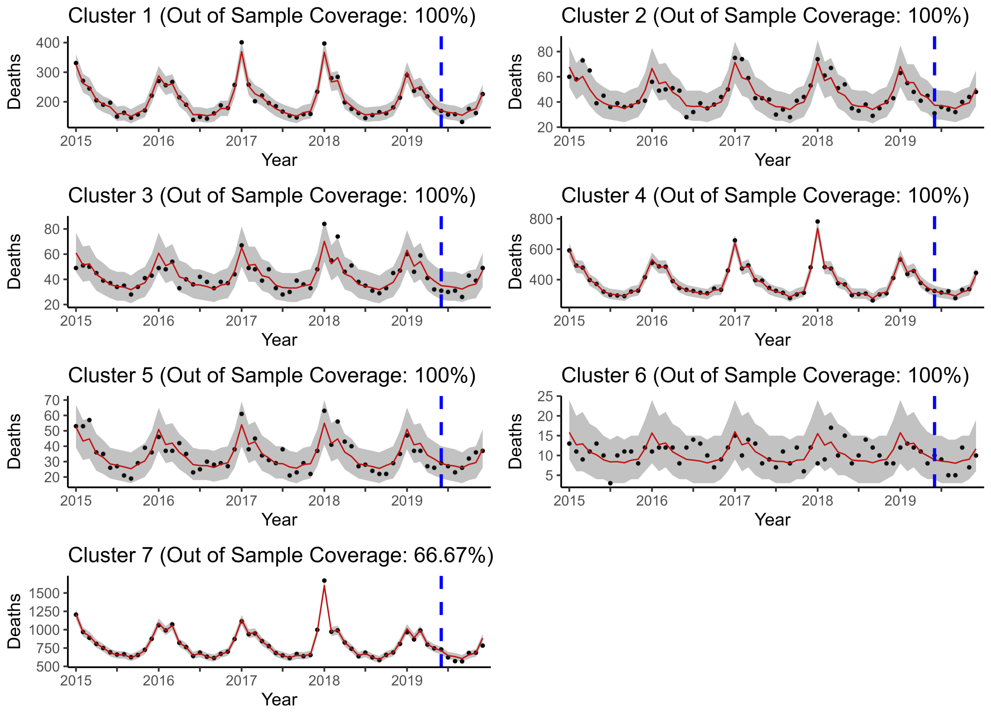

Supplement: S14 Appendix — (PDF) [file pone.0348787.s014.pdf]
