# Supplementary material for: KGR-SKATER: Spatially clustered kernel graph regression for counting processes
Source: PLoS One. 2026 May 20;21(5):e0348787. doi: 10.1371/journal.pone.0348787 (PMC13189423; doi:10.1371/journal.pone.0348787)
Supplement: S15 Appendix — (PDF) [file pone.0348787.s015.pdf]

S15 Appendix for KGR-SKATER: Spatially Clustered Kernel Graph Regression for Counting Processes

Jeffrey Wu[1,,¤*], Gareth W. Peters[1,¤,*], Alex Franks[1,¤,*],

1 Department of Statistics & Applied Probability, UCSB, Santa Barbara, California, USA

¤5607 South Hall Santa Barbara, CA 93106-2014, USA
* jeffreywu@pstat.ucsb.edu,garethpeters@pstat.ucsb.edu,afranks@pstat.ucsb.edu

S15: Additional out of sample forecast performance tables

In addition to the scaled RMSPE metric, some additional forecast accuracy metrics were calculated to evaluate out of sample fit at each cluster c. This appendix presents each of these performance metrics in their own table.

S15.1 MAE

Mean absolute error (MAE) is a measure of the average size of the mistakes in a collection of predictions, without taking their direction into account.

$$MAE_c = \frac{1}{h_{max}} \sum_{h=1}^{h_{max}} |\hat{\lambda}^{obs}_{c,t+h} - \hat{\lambda}_{c,t+h}|$$

Table S15.1. Out-of-sample MAE for each model.

	**Reference Models**		**Proposed Models**				
	#2	#3	#1	#2	#3	#4	#5
**2 clusters**							
Cluster 1	3.295	5.156	**2.604**	3.469	2.696	2.640	3.070
Cluster 2	9.9213	113.3725	17.285	12.318	11.590	13.179	**7.871**
**7 clusters**							
Cluster 1	1.410	2.623	**1.180**	1.278	1.265	1.248	1.339
Cluster 2	2.593	7.090	2.404	2.416	2.407	**2.389**	2.413
Cluster 3	6.274	48.180	8.390	6.234	6.171	6.514	**6.148**
Cluster 4	5.753	27.068	**4.715**	5.439	5.593	5.393	5.088
Cluster 5	1.640	2.994	1.618	1.613	**1.609**	1.614	1.610
Cluster 6	1.269	**1.159**	1.278	1.273	1.272	1.257	1.245
Cluster 7	10.270	44.450	12.213	10.579	10.318	**9.812**	10.314

Notice that the proposed models tend to have slightly better forecast accuracy than the reference models. These results were obtained using SKATER's minimum population constraint.

S15.2 MASE

Mean absolute scaled error (MASE) is a measure of the accuracy of forecasts. It is the mean absolute error of the forecast values, divided by the mean absolute error of the in-sample one-step naive forecast.

$$MASE_c = \frac{\frac{1}{n}\sum_{t=1}^{n} |\hat{\lambda}_{c,t+h}^{obs} - \hat{\lambda}_{c,t+h}|}{\frac{1}{n-1}\sum_{t=2}^{n} |\hat{\lambda}_{c,t+h}^{obs} - \hat{\lambda}_{c,t+h-1}^{obs}|}$$

Table S15.2. Out-of-sample MASE for each model.

	**Reference Models**		**Proposed Models**				
	#2	#3	#1	#2	#3	#4	#5
**2 clusters**							
Cluster 1	0.1399	0.62286	0.2041	0.2133	0.0870	0.1575	0.2239
Cluster 2	0.0265	0.2883	0.0497	0.0326	0.0293	0.0352	0.0242
**7 clusters**							
Cluster 1	0.1007	0.2137	**0.0756**	0.0920	0.0903	0.0872	0.1018
Cluster 2	0.1108	0.3050	0.1017	0.1031	0.1027	**0.1017**	0.1030
Cluster 3	0.1150	0.8651	0.1497	0.1124	**0.1104**	0.1155	0.1126
Cluster 4	0.3877	1.6517	**0.2780**	0.3713	0.3908	0.3721	0.3611
Cluster 5	0.4052	0.8629	**0.3632**	0.3855	0.3870	0.3912	0.3853
Cluster 6	0.1186	**0.1133**	0.1206	0.1193	0.1194	0.1175	0.1166
Cluster 7	**0.0525**	0.3393	0.0970	0.0682	0.0657	0.0576	0.0646

Notice that the proposed models tend to have slightly better forecast accuracy than the reference models. These results were obtained using SKATER's minimum population constraint.

S15.3 MAPE

Mean absolute percentage error (MAPE), also known as mean absolute percentage deviation (MAPD), is a measure of prediction accuracy of a forecasting method in statistics, expressing accuracy as a ratio.

$$MAPE_c = \frac{100}{n} \sum_{t=1}^{n} |\frac{\hat{\lambda}^{obs}_{c,t+h} - \hat{\lambda}_{c,t+h}}{\hat{\lambda}^{obs}_{c,t+h}}|$$

Table S15.3. Out-of-sample MAPE for each model.

	Reference Models		Proposed Models				
	#2	#3	#1	#2	#3	#4	#5
**2 clusters**							
Cluster 1	0.0422	0.0697	**0.0328**	0.0441	0.0342	0.0334	0.0397
Cluster 2	0.0082	0.0899	0.0144	0.0099	0.0094	0.0109	**0.0067**
**7 clusters**							
Cluster 1	0.0383	0.0738	**0.0317**	0.0346	0.0343	0.0339	0.0366
Cluster 2	0.0682	0.2021	0.0628	0.0634	0.0632	**0.0627**	0.0634
Cluster 3	0.0199	0.1495	0.0241	0.0183	0.0182	0.0192	**0.0181**
Cluster 4	0.0365	0.1656	**0.0286**	0.0338	0.0350	0.0335	0.0318
Cluster 5	0.0616	0.1132	**0.0606**	0.0608	0.0607	0.0609	0.0607
Cluster 6	0.1315	0.1391	0.1330	0.1325	0.1325	0.1308	**0.1295**
Cluster 7	0.0142	0.0652	0.0177	0.0150	0.0146	**0.0139**	0.0147

Notice that the proposed models tend to have slightly better forecast accuracy than the reference models. These results were obtained using SKATER's minimum population constraint.
