# Supplementary material for: KGR-SKATER: Spatially clustered kernel graph regression for counting processes
Source: PLoS One. 2026 May 20;21(5):e0348787. doi: 10.1371/journal.pone.0348787 (PMC13189423; doi:10.1371/journal.pone.0348787)
Supplement: S16 Appendix — (PDF) [file pone.0348787.s016.pdf]

S16 Appendix for KGR-SKATER: Spatially Clustered Kernel Graph Regression for Counting Processes

Jeffrey Wu[1,¤,*], Gareth W. Peters[1,¤,*], Alex Franks[1,¤,*],

1 Department of Statistics & Applied Probability, UCSB, Santa Barbara, California, USA

¤5607 South Hall Santa Barbara, CA 93106-2014, USA
* jeffreywu@pstat.ucsb.edu,garethpeters@pstat.ucsb.edu,afranks@pstat.ucsb.edu

S16: Fitting proposed models with and without monthly fixed effects

This appendix includes posterior predictive plots illustrating the difference in model fits when monthly fixed effects are included versus not. The posterior predictive plots for $\mathcal{M}_4$ and $\mathcal{M}_5$ with and without monthly fixed effects are shown below:

Fig S16.1. Posterior predictive plots for $\mathcal{M}_4$ with monthly fixed effects. It appears that KGR-SKATER models can fit the mortality data well either way.

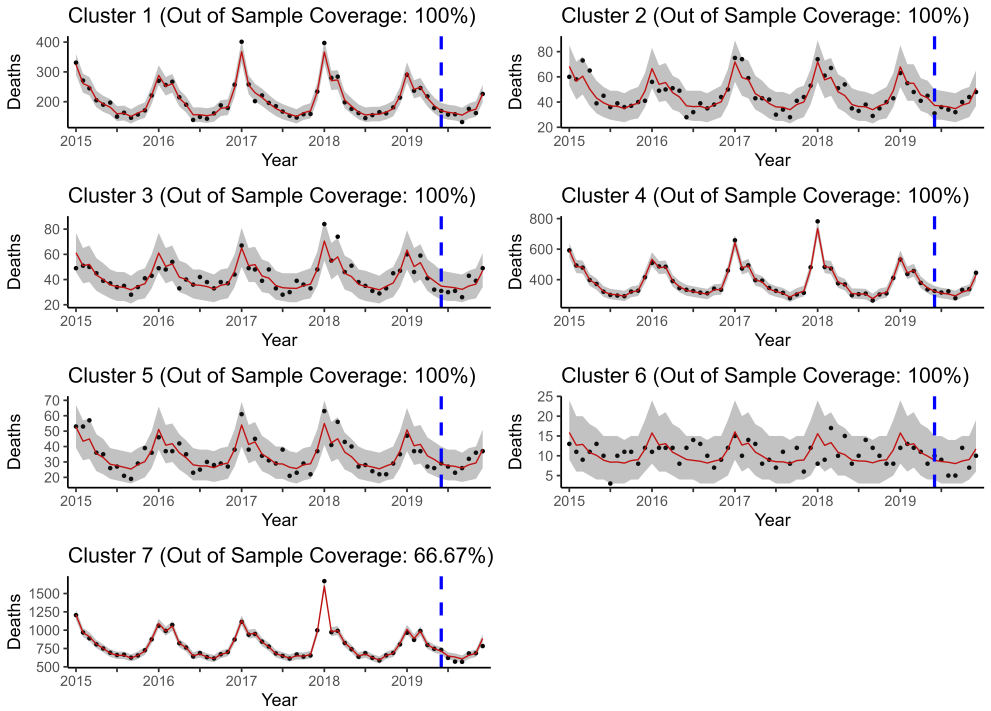

Fig S16.2. Posterior predictive plots for $\mathcal{M}_4$ without monthly fixed effects. It appears that KGR-SKATER models can fit the mortality data well either way.

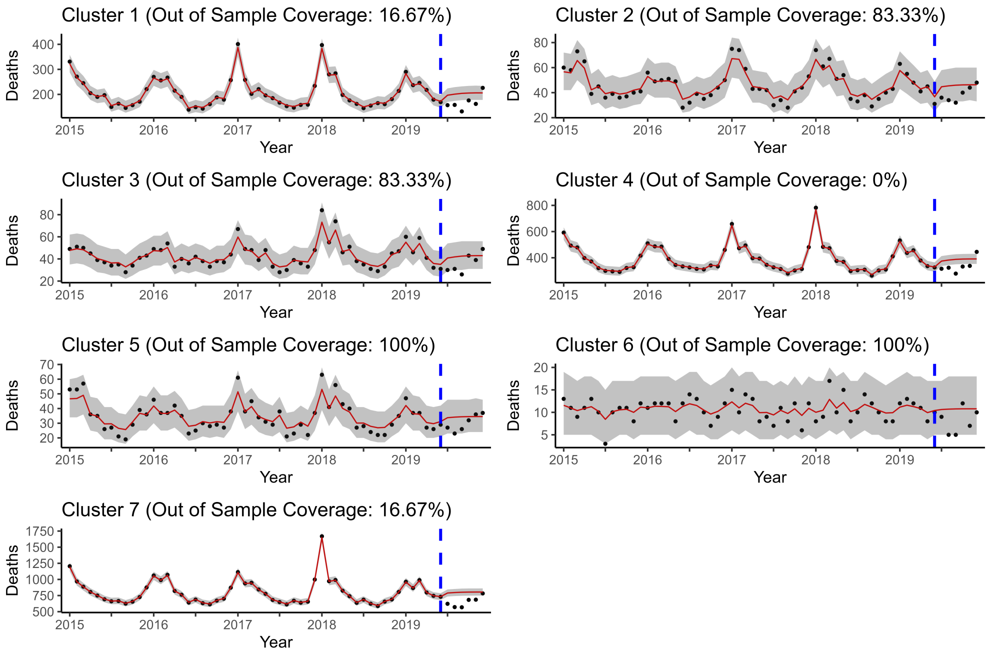

Fig S16.3. Posterior predictive plots for $\mathcal{M}_5$ with monthly fixed effects. It appears that KGR-SKATER models can fit the mortality data well either way.

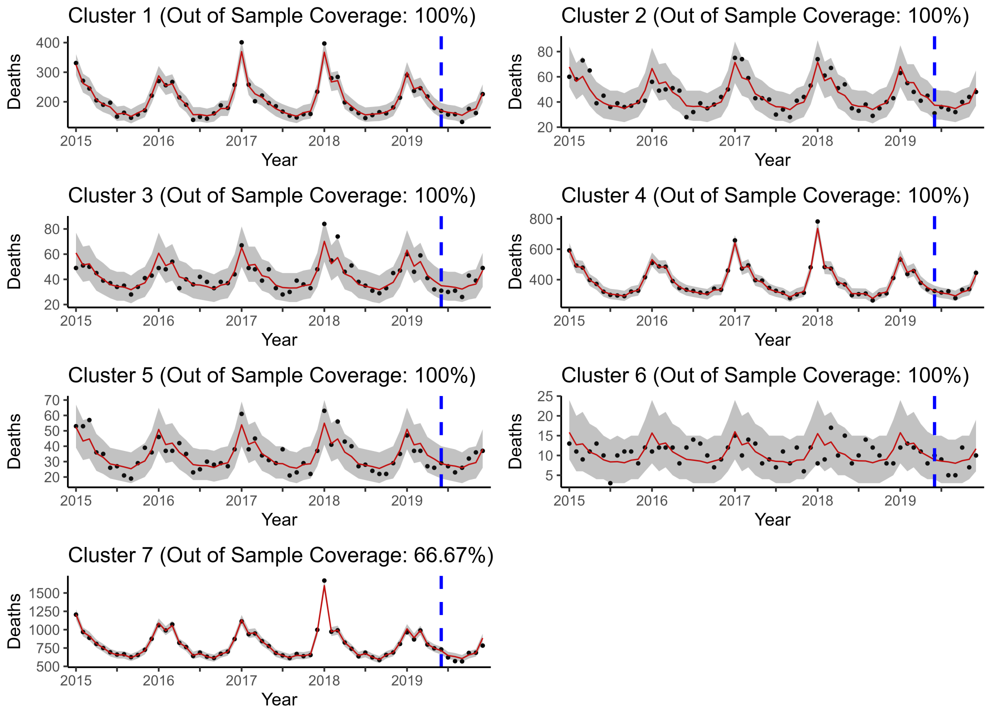

Fig S16.4. Posterior predictive plots for $\mathcal{M}_5$ without monthly fixed effects. It appears that KGR-SKATER models can fit the mortality data well either way.

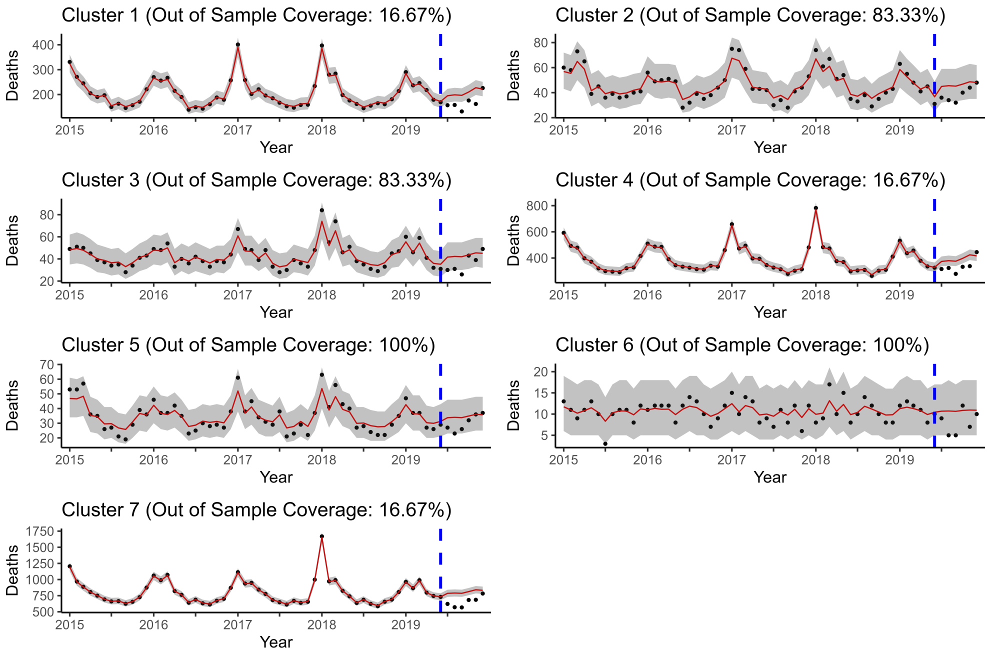

The plots above illustrate that including these fixed effects leads to a better fitting model with respect to prediction accuracy and coverage. The time series predicted by the model without fixed effects tend towards the mean and are generally smoother. The credible interval bands are about the same width as those for the model with the monthly fixed effects, but it appears that they do not cover the observed data quite as well. If there is strong temporal dependence as is the case in the application study, including temporal fixed effects is probably more appropriate.
