# Supplementary material for: KGR-SKATER: Spatially clustered kernel graph regression for counting processes
Source: PLoS One. 2026 May 20;21(5):e0348787. doi: 10.1371/journal.pone.0348787 (PMC13189423; doi:10.1371/journal.pone.0348787)
Supplement: S17 Appendix — (PDF) [file pone.0348787.s017.pdf]

S17 Appendix for KGR-SKATER: Spatially Clustered Kernel Graph Regression for Counting Processes

Jeffrey Wu[1,,¤*], Gareth W. Peters[1,¤,*], Alex Franks[1,¤,*],

1 Department of Statistics & Applied Probability, UCSB, Santa Barbara, California, USA

¤5607 South Hall Santa Barbara, CA 93106-2014, USA
* jeffreywu@pstat.ucsb.edu,garethpeters@pstat.ucsb.edu,afranks@pstat.ucsb.edu

S17: Rolling window forecast exercise

This appendix contains the calculated MAE, MASE, MAPE, and RMSPE for the reference and proposed models from the rolling window forecasting exercise.

Table S17.1. Rolling forecast metrics by model and forecast horizon h.

	Reference Model				Proposed Model			
h	MAE	MASE	MAPE	RMSPE	MAE	MASE	MAPE	RMSPE
1	18.229	0.021	0.089	25.498	15.514	0.027	0.093	21.952
2	7.600	0.019	0.049	12.803	9.314	0.019	0.055	15.917
3	3.000	0.027	0.043	3.797	2.429	0.027	0.040	3.332
4	3.400	0.012	0.024	6.187	4.257	0.011	0.028	7.042
5	4.171	0.066	0.035	5.475	4.457	0.065	0.036	5.770
6	2.571	0.040	0.040	3.141	2.286	0.038	0.040	2.894
7	4.971	0.084	0.049	6.975	4.914	0.083	0.050	6.876
8	3.629	0.049	0.065	4.873	3.571	0.047	0.067	4.669
9	5.829	0.093	0.080	8.025	5.400	0.090	0.078	7.478
10	1.714	0.042	0.035	2.141	1.514	0.040	0.037	1.752
11	4.657	0.080	0.062	5.827	4.657	0.078	0.062	5.827
12	9.371	0.004	0.058	17.473	9.086	0.004	0.057	17.400
13	18.229	0.013	0.089	25.498	16.657	0.015	0.094	23.444
14	7.600	0.016	0.049	12.803	8.457	0.016	0.054	14.322
15	3.000	0.028	0.043	3.797	2.714	0.029	0.043	3.532
16	3.400	0.013	0.024	6.187	3.829	0.011	0.028	6.425
17	4.171	0.066	0.035	5.475	4.314	0.064	0.035	5.612
18	2.571	0.039	0.040	3.141	2.429	0.037	0.040	2.996
19	4.971	0.084	0.049	6.975	4.971	0.081	0.049	6.975
20	3.629	0.048	0.065	4.873	3.629	0.045	0.065	4.873
21	5.829	0.091	0.080	8.025	5.686	0.088	0.079	7.876
22	1.714	0.041	0.035	2.141	1.571	0.039	0.034	1.908
23	4.800	0.080	0.067	5.853	4.657	0.077	0.062	5.827
24	9.371	0.003	0.058	17.473	9.371	0.004	0.061	17.463

The forecast metrics for $\mathcal{M}_4$ are a little better than those produced by $\mathcal{M}_2^R$. The posterior predictive means estimated by each model were almost the same in most cases, give or take one or two deaths.

As stated in the main paper, the rolling forecast performance for models $\mathcal{M}_4$ and $\mathcal{M}_2^R$ are more or less the same. $\mathcal{M}_4$ performs slightly better with respect to accuracy while $\mathcal{M}R_2$ performs slightly better with respect to uncertainty quantification. This is likely due to the fact that the predictions made by both models rely heavily on the monthly fixed effects.
