# Supplementary material for: KGR-SKATER: Spatially clustered kernel graph regression for counting processes
Source: PLoS One. 2026 May 20;21(5):e0348787. doi: 10.1371/journal.pone.0348787 (PMC13189423; doi:10.1371/journal.pone.0348787)
Supplement: S18 Appendix — (PDF) [file pone.0348787.s018.pdf]

S18 Appendix for KGR-SKATER: Spatially Clustered Kernel Graph Regression for Counting Processes

Jeffrey Wu[1,,¤*], Gareth W. Peters[1,¤,*], Alex Franks[1,¤,*],

1 Department of Statistics & Applied Probability, UCSB, Santa Barbara, California, USA

¤5607 South Hall Santa Barbara, CA 93106-2014, USA
* jeffreywu@pstat.ucsb.edu,garethpeters@pstat.ucsb.edu,afranks@pstat.ucsb.edu

S18: Fitting a KGR-SKATER model with Negative Binomial likelihood

This appendix explores fitting a KGR-SKATER with a Negative Binomial instead of a Poisson likelihood due to the fact that the response exhibits signs of overdispersion.

S18.1 In sample fit

Fig S18.1. Posterior predictive mean and credible interval bands estimated by Negative Binomial KGR-SKATER model with the same covariance structure as $\mathcal{M}_4$. The fits are similar with the exception of slightly worse coverage. This suggests that the original proposed KGR-SKATER models may be overfitting the data.

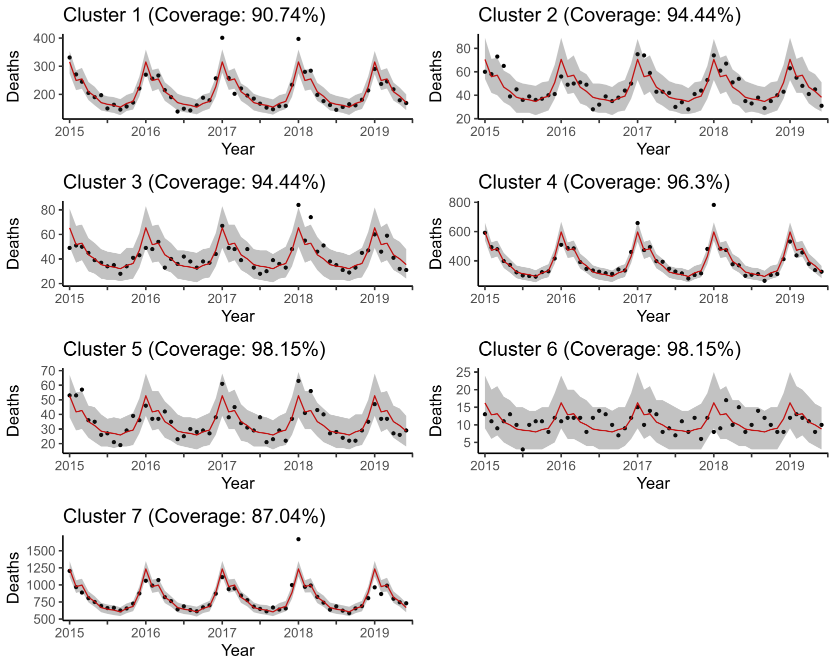

The WAIC for the Negative Binomial KGR-SKATER model equivalent of $\mathcal{M}_4$ is 2926.256. Recall that $\mathcal{M}_4$ had a lower WAIC (2808.770), which is why it was used for

the application study analysis. The in sample RMSPE values for the Negative Binomial KGR-SKATER model were about the same as well (0.2319, 0.2327, 0.2328, 0.2323, 0.2325, 0.2332, 0.2333) for clusters 1-7, respectively.

S18.2 Out of sample fit

Fig S18.2. Posterior predictive mean and credible interval bands estimated by Negative Binomial KGR-SKATER model with the same covariance structure as $\mathcal{M}_4$. The fits are similar but the coverage improves slightly because one additional out of sample observation in cluster 7 is captured by the credible interval bands.

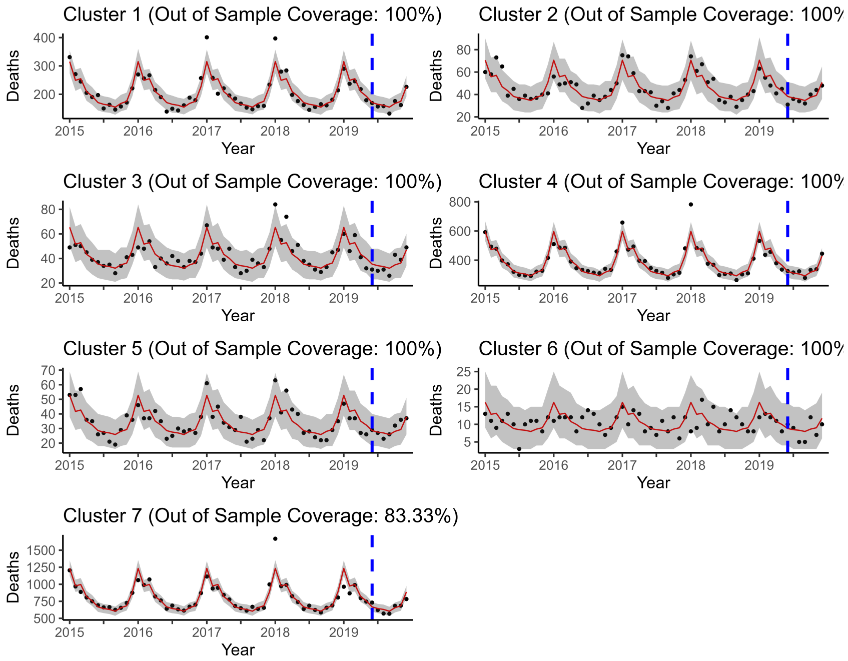

In comparison to $\mathcal{M}_4$, the Negative Binomial KGR-SKATER model has a slightly worse overall coverage (0.9452 vs 0.9929) and about the same level of forecasting accuracy for the six months ahead forecast (0.0360, 0.0726, 0.0419, 0.0212, 0.0665, 0.1491, 0.0166) for clusters 1-7, respectively.

S18.3 Rolling window forecast

Fig S18.3. Rolling window forecasts produced by Negative Binomial KGR-SKATER model with the same covariance structure as $\mathcal{M}_4$. The fixed effects still dominate INLA's estimates. The out of sample coverage improves for clusters 1,4,6, and 7.

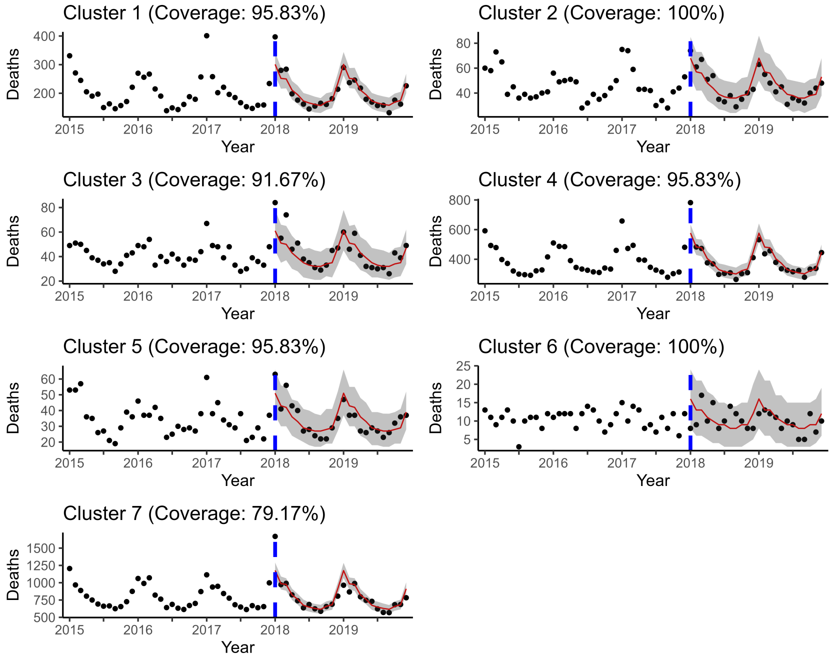

The Negative Binomial KGR-SKATER model does produce slightly wider 95% credible interval bands. This suggests that even though it was not favored by WAIC, it may be a more appropriate model choice for the application study. Additionally, it has about the same level of forecasting accuracy as the original proposed models, as shown in the table of forecast metrics below.

Table S18.4. Rolling forecast metrics for each forecast horizon point h.

	$\mathcal{M}_4$ (NB)				$\mathcal{M}_4$			
h	MAE	MASE	MAPE	RMSPE	MAE	MASE	MAPE	RMSPE
1	15.657	0.027	0.093	22.108	15.514	0.027	0.093	21.952
2	9.029	0.019	0.054	15.476	9.314	0.019	0.055	15.917
3	2.571	0.027	0.043	3.454	2.429	0.027	0.040	3.332
4	3.971	0.011	0.028	6.555	4.257	0.011	0.028	7.042
5	4.314	0.065	0.035	5.612	4.457	0.065	0.036	5.770
6	2.286	0.038	0.039	2.721	2.286	0.038	0.040	2.894
7	4.771	0.083	0.046	6.993	4.914	0.083	0.050	6.876
8	3.714	0.048	0.071	4.510	3.571	0.047	0.067	4.669
9	4.971	0.090	0.077	6.949	5.400	0.090	0.078	7.478
10	1.429	0.040	0.034	1.727	1.514	0.040	0.037	1.752
11	4.800	0.079	0.070	5.685	4.657	0.078	0.062	5.827
12	9.086	0.004	0.057	17.400	9.086	0.004	0.057	17.400
13	16.229	0.015	0.094	22.849	16.657	0.015	0.094	23.444
14	8.600	0.016	0.054	14.681	8.457	0.016	0.054	14.322
15	2.714	0.029	0.043	3.532	2.714	0.029	0.043	3.532
16	3.829	0.011	0.028	6.425	3.829	0.011	0.028	6.425
17	4.314	0.064	0.035	5.612	4.314	0.064	0.035	5.612
18	2.286	0.037	0.039	2.721	2.429	0.037	0.040	2.996
19	5.057	0.082	0.050	7.131	4.971	0.081	0.049	6.975
20	3.714	0.046	0.071	4.510	3.629	0.045	0.065	4.873
21	5.400	0.088	0.078	7.478	5.686	0.088	0.079	7.876
22	1.571	0.039	0.034	1.908	1.571	0.039	0.034	1.908
23	4.800	0.078	0.070	5.685	4.657	0.077	0.062	5.827
24	9.086	0.003	0.057	17.400	9.371	0.004	0.061	17.463

The forecast metrics for $\mathcal{M}_4$ are a little better than those produced by $\mathcal{M}_2^R$. The posterior predictive means estimated by each model were almost the same in most cases, give or take one or two deaths.
