# Supplementary material for: KGR-SKATER: Spatially clustered kernel graph regression for counting processes
Source: PLoS One. 2026 May 20;21(5):e0348787. doi: 10.1371/journal.pone.0348787 (PMC13189423; doi:10.1371/journal.pone.0348787)
Supplement: Attachment — Submitted filename: Review of KGR-SKATER.pdf [file pone.0348787.s019.pdf]

Review of KGR-SKATER: Spatially clustered kernel graph regression

This manuscript introduces the KGR-SKATER framework, a novel methodology for modeling high-dimensional, non-Gaussian spatiotemporal data. The framework aims to provide an interpretable and computationally efficient approach to model complex spatiotemporal dependencies in data. The methodology proposed includes the following ingredients: (1) Spatial Clustering. The SKATER algorithm is used to group spatial units into clusters based on a selected feature (e.g., socioeconomic data--Social Deprivation Index - SDI), reducing dimensionality and revealing spatial heterogeneity. (2) Graph-Based Modeling: A spatial dependence graph is estimated using the HUGE package and graphical LASSO, which is then transformed into a graph Laplacian and filtered to smooth high-frequency components. (3) The temporal dependencies are modeled using kernel regression with various kernel functions (e.g., locally periodic, damped period, and lagged time series kernels) to capture nonstationary and dynamic patterns. (4) Spatial and temporal dependencies are combined using a Kronecker product to produce a separable spatiotemporal covariance matrix. (5) The framework uses Integrated Nested Laplace Approximation (INLA) for efficient Bayesian inference, enabling rapid model fitting and hyperparameter optimization.

The KGR-SKATER framework was applied to model respiratory-related deaths across California from 2015 to 2019, using SDI for spatial dependence and air quality data for temporal dependence. SKATER clustering reduced California's 58 counties into seven spatial clusters based on SDI similarity and spatial contiguity. The model was compared with three reference models (Poisson GLMM, Besag-York-Mollie model, and LGCP with time kernel). KGR-SKATER demonstrated comparable predictive accuracy to reference models but provided better uncertainty quantification, as evidenced by wider credible intervals and higher frequentist coverage rates. The KGR-SKATER models effectively captured volatile periodicity and amplitude in time series data. The proposed models showed slightly better out-of-sample forecast accuracy and improved coverage compared to reference models.

The manuscript is overall well-written and enjoyable to read, the research is supported by several Appendices, and the topic is of interest for researchers in different scientific fields. It is apparent that the authors have made a considerable effort to present a difficult and complex subject. At the same time, there are certain aspects of the work that could be improved before the manuscript is accepted.

First of all, since KGR-SKATER incorporates several methodologies from spatiotemporal statistics, the innovation of this study should be more clearly stated in the context of the current literature. The authors mention in the Introduction that this is a novel procedure, but since KGR-SKATER draws from various methods, it would be useful to clarify which are the most innovative elements of this approach---according to the authors’ assessment.

The KGR-SKATER framework is highly complex, involving multiple steps such as spatial clustering, graph estimation, kernel regression, and Bayesian inference. This complexity may make it challenging for practitioners without advanced technical expertise to implement the methodology and it may limit the impact of the study. Figure 1 is a powerful tool for presenting this complexity. I would propose enhancing Figure 1 or complementing it with a second figure that will help to convey the different steps of the method. For example, the caption of Fig. 1 could clarify the covariates and why they are handled differently. Input and response variables should also appear and be identified. The steps where the graph Laplacian is used should appear in the diagram. Various diagram elements refer to clustering; the clustering method should be mentioned for each of these steps. The steps that involve the temporal kernel and the Kronecker product construction should be identified in the diagram, etc. Some of the boxes (in Fig. 1 or in a complementary figure) could be replaced/complemented with an illustrative graph.

The framework relies on computationally intensive methods, such as Integrated Nested Laplace Approximation (INLA) and grid search for hyperparameter optimization. While INLA is faster than traditional MCMC methods, the grid search can still be computationally expensive, especially for large datasets. The Discussion section could present a high-level analysis of potential computational bottlenecks. If possible, estimates of how the computational complexity and memory requirements scale with data size and covariate-space dimension should be given.

The study compares the proposed models with three reference models, which may not fully represent the breadth of existing spatiotemporal modeling approaches. Including more diverse benchmarks could strengthen the claims of superiority. While this is not recommended action for the current manuscript, at least some discussion of other relevant methodologies could be presented in the Discussion section.

Although the paper explores various kernel structures, the proposed models are relatively similar, and the differences between them are minor. A more diverse set of kernel configurations could provide deeper insights into the framework's flexibility. An explicit equation is given only for the combination of the periodic and RBF kernels. I do not suggest pursuing analysis of different kernels in this study, since this would lead to an even longer manuscript; however, at least some comments can be included in the Discussion section regarding the applicability and potential impact on the results of commonly used kernel such as the flexible Mat’ern functions which feature a smoothness index parameter (e.g., Rasmussen & Williams, Gaussian processes for Machine Learning, 2006). For periodic kernels since I am not aware of many choices, I would suggest considering in a future study the Brownian oscillator model and its spatiotemporal extension:

D. T. Hristopulos, "Non-Separable Covariance Kernels for Spatiotemporal Gaussian Processes Based on a Hybrid Spectral Method and the Harmonic Oscillator," *IEEE Transactions on Information Theory*, vol. 70, no. 2, pp. 1268-1283, Feb. 2024, doi: 10.1109/TIT.2023.3321215

While the KGR-SKATER framework is described as interpretable, the combination of spatial clustering, graph-based modeling, and kernel regression may make it difficult for non-experts to understand the underlying mechanisms and interpret the results. The paper could benefit from detailed guidance on how practitioners can implement the framework in real-world scenarios, such as handling missing data, selecting optimal kernel structures, and addressing computational challenges.

The following comment is not critical, but it could be useful if the authors can provide some guidance in this respect: The KGR-SKATER framework heavily relies on specific software packages (e.g., R packages like SKATER, HUGE, and INLA). This dependency may limit accessibility for researchers who use other programming languages. Are there any options for researchers who work with Python and Matlab?

The paper does not explicitly discuss potential limitations of the KGR-SKATER framework, such as its sensitivity to hyperparameter choices, assumptions about spatial and temporal dependencies, or challenges in scaling to larger datasets. Such potential issues could be discussed in the Discussion section. In addition, comments regarding how the model could be used for actionable insights in public health or other fields could also be included to strengthen the appeal of the manuscript.

In relation to recent developments in the literature, it is worth noting that several studies have introduced computationally efficient kernel-based approaches for space–time interpolation of irregularly distributed data. These methods construct graphs based on local neighborhood structures, which in turn yield sparse precision matrices:

[1] D. T. Hristopulos (2015). Stochastic Local Interaction (SLI) model: Bridging machine learning and geostatistics, *Computers & Geosciences* Vol. 85, pp. 26-37

[2] D. T. Hristopulos, V. D. Agou (2020). Stochastic local interaction model with sparse precision matrix for space–time interpolation, *Spatial Statistics*, Vol. 40, p. 100403.

The models used in these applications likewise incorporate Dirichlet-type energies within spatial or spatiotemporal frameworks. However, they adopt a distinct strategy for parameter estimation, relying primarily on likelihood maximization—facilitated by the sparsity of the precision matrix—together with cross-validation procedures.

In addition, while KGR-SKATER focuses on constructing separable space-time covariance kernels using the Kronecker product of spatial and temporal components, [2] uses a Kronecker product of temporal and spatial components to construct a separable precision matrix; the latter corresponds to a non-separable covariance structure.

Some Technical Comments:

The authors mention that they use longitude and latitude as coordinates. How are the distances calculated? Are Euclidean distances used based on a geographical projection? Or are geodesic distances used instead? In the latter case, what has been done to ensure that the spatial covariance kernels are admissible on the sphere?

In Section 2.1, the authors present a form for the locally periodic kernel. I do not fully understand what ***x*** and ***x***' represent in this equation. According to the earlier definition of the covariates, it seems that ***x*** and ***x***' represent covariates (not spatial locations). In such a case, what is the physical meaning of the Euclidean distance implied in **|** ***x*** - ***x***'**|**? And shouldn't the ***x*** values be scaled in order to calculate such distances?

Furthermore, what drives the selection of a quasi-periodic kernel for covariate dependence? If on the other hand ***x*** and ***x***' refer to locations instead of covariates, the notation should be corrected.

The mortality data used in the application study was censored to protect privacy, and the authors used the Expectation-Maximization (EM) algorithm for imputation. While this approach is reasonable, the imputation process may introduce bias or uncertainty. Can you comment on such potential effects?
