# Supplementary material for: KGR-SKATER: Spatially clustered kernel graph regression for counting processes
Source: PLoS One. 2026 May 20;21(5):e0348787. doi: 10.1371/journal.pone.0348787 (PMC13189423; doi:10.1371/journal.pone.0348787)
Supplement: Attachment — Submitted filename: Response_to_Reviewers.pdf [file pone.0348787.s021.pdf]

Jeffrey Wu
PhD Candidate
Department of Statistics and Applied Probability
University of California Santa Barbara

March 24, 2026

Revision One: Submission to PlosOne

Dear Editors and Reviewers,

My co-authors and I thank you for your time and consideration of our manuscript titled *" KGR-SKATER: Spatially clustered kernel graph regression for counting processes "*.

We thank the editor and reviewer for their careful reading of the manuscript and for their constructive comments. We are pleased that the reviewer found the manuscript technically sound and interesting. We have revised the manuscript extensively to address all comments and suggestions.

Please find enclosed below the detailed point-by-point response to the reviewers' suggestions and comments. We aim to provide a careful and articulate response that addresses their useful suggestions and, where appropriate, we make changes to update the manuscript. We will alert the reviewers to such changes in the response letter and highlight our reasoning for such changes when required. We believe that in doing so, we have provided sufficient amendments to make the paper suitable for publication in PlosOne.

Sincerely,

Jeffrey Wu & Prof. Gareth W. Peters & Dr. Alex Franks

University of California Santa Barbara
CA 93106, California, USA
Email: jeffreywu@pstat.ucsb.edu

1 Response to Reviewers' comments

In the following, we outline the individual reviewer comments and questions, and the steps we took to address the comments or suggestions when required.

1.1 Figures/Tables

```
Copyright status of included maps:
We note that Figures 3, S1, S2, S5 and S8 in your submission contain
map images which may be copyrighted.
```

Response: All of the maps included in figures for the main and supplemental documents were generated by the authors using publicly available geographic boundary data from the U.S. Census Bureau (`https://catalog.data.gov/dataset/tiger-line-shapefile-2016-state-california-current-place-state-based`). These datasets are produced by the U.S. federal government and are in the public domain (17 U.S.C. §105), and therefore do not require permission for reuse or publication.

The figures were created by the authors in R using the maps, sf, and ggplot2 packages based on these public-domain shapefiles. Appropriate attribution to the U.S. Census Bureau TIGER/Line dataset has been added to the figure captions in the revised manuscript.

```
Remake Table 1 and Figures 1 and 10:
Table 1 and Figures 1 and 10 are difficult to read.
```

Response: Table 1 and Figure 10 have been remade to be larger so they are more legible. Figure 1 has been reformatted and enhanced to be more detailed and legible.

1.2 Reviewer #1: Comments, Suggestions and Questions

```
Reviewer #1:
The innovation of the study should be clarified since the framework
combines several existing methodologies.
```

Response: We agree with the reviewer that the novelty of the KGR-SKATER framework should be more clearly articulated. We have revised the Introduction to explicitly highlight the methodological contributions of the proposed framework. In particular, we now emphasize three key innovations:

1. The integration of spatial clustering with data-driven graph estimation.

University of California Santa Barbara
CA 93106, California, USA
Email: jeffreywu@pstat.ucsb.edu

2. The construction of a graph-filtered Gaussian process prior via the Kronecker product of spatial and temporal components.
3. Efficient estimation of the resulting model using INLA instead of MCMC.

These clarifications have been added to the Introduction.

```
Reviewer #1:
The framework is complex and may be difficult to implement.  Figure
1 could be improved.
```

Response: We appreciate this suggestion. Figure 1 has been revised and reformatted to better illustrate the modeling workflow and the relationships between data inputs, clustering, graph estimation, kernel construction, and the final spatiotemporal model. The figure caption has also been expanded to explain each stage of the procedure.

```
Reviewer #1:
Discussion of computational bottlenecks and scalability should be
added.
```

Response: We have added a new subsection in the Discussion titled "Computational Complexity and Scalability", which provides an overview of the computational cost of each stage of the framework (clustering, graph estimation, kernel construction, and INLA inference) and discusses how the dimensionality reduction achieved through clustering improves scalability.

```
Reviewer #1:
Other spatiotemporal modeling approaches could be discussed.
```

Response: We have expanded the Introduction section to include references to additional kernel-based spatiotemporal models, including stochastic local interaction models proposed by Hristopulos (2015) and Hristopulos & Agou (2020). The revised text explains how these approaches differ from the proposed framework.

```
Reviewer #1:
More discussion of alternative kernels would be useful.
```

Response: We have added a discussion of alternative kernel functions, including the Matérn family and recently proposed non-separable spatiotemporal kernels. We agree that exploring these kernels would be an interesting direction for future research.

University of California Santa Barbara
CA 93106, California, USA
Email: jeffreywu@pstat.ucsb.edu

```
Reviewer #1:
Guidance for practitioners would improve the manuscript.
```

Response: A new subsection titled "Practical Implementation Considerations" has been added (see Section 6.1). This section outlines how practitioners may select clustering parameters, kernel structures, and hyperparameters when applying the framework.

```
Reviewer #1:
The reviewer suggests discussing potential limitations of the
KGR-SKATER framework and providing more context on how the model
may produce actionable insights in applied settings.
```

Response: We thank the reviewer for this insightful suggestion. In response, we have revised and expanded the Discussion section to more clearly articulate the key modeling design choices underpinning the KGR-SKATER framework and their implications in applied settings. In particular, we clarify that the use of a separable spatiotemporal covariance structure is a deliberate structural decomposition that enables the model to disentangle and interpret distinct sources of spatial and temporal variation, rather than a restrictive assumption.

We also emphasize that the specification of components such as the number of SKATER clusters, the graph learning procedure, the spectral filtering mechanism, and the temporal kernel design are not merely tuning parameters, but represent interpretable modeling choices that can be guided by domain knowledge to reflect application-specific dependence structures.

Furthermore, we have expanded the discussion on how the resulting structured dependence representation improves uncertainty quantification, which in turn facilitates more reliable forecasting and supports actionable decision-making in applied contexts such as public health and policy planning. Finally, we comment on computational considerations in terms of scalability, framing them within the broader context of model resolution and structural complexity, which can be adjusted in a principled manner depending on the application.

```
Reviewer #1:
Clarify how distances are calculated using latitude and longitude.
```

Response: We have clarified that spatial distances are computed using great-circle distances derived from geographic coordinates.

Reviewer #1:
Clarify the meaning of the variables x and x' in the kernel function.

University of California Santa Barbara
CA 93106, California, USA
Email: jeffreywu@pstat.ucsb.edu

Response: The notation has been clarified in Section 2.1. We now explicitly state that x and x' represent vectors of surrogate covariates rather than spatial locations, and that these variables are standardized prior to computing Euclidean distances.

```
Reviewer #1:
Discuss possible bias introduced by EM imputation.
```

Response: We have added a paragraph discussing the potential effects of EM-based imputation of censored mortality counts and the extent to which this procedure may introduce additional uncertainty.

```
Reviewer #1:
The framework relies heavily on R packages.
```

Response: We have added a new subsection in the Discussion titled "Software Accessibility", which explains that equivalent implementations are available in other programming environments (e.g., Python and MATLAB) and that the methodology itself is not restricted to R.

2 Additional changes

In response to editorial requirements, we have:

- Improved figure resolution.
- Replaced map figures with publicly available data sources.
- Reformatted Table 1.
- Added direct links to the data sources.
- Provided a GitHub repository containing the code used in the analysis.

We believe that these revisions have substantially improved the clarity and accessibility of the manuscript, and we thank the reviewer again for their helpful suggestions.

Sincerely,
The Authors

University of California Santa Barbara
CA 93106, California, USA
Email: jeffreywu@pstat.ucsb.edu
